# Benchmarking Large Language Models in Acute-on-Chronic Pancreatitis: An Exploratory Study

**DOI:** 10.3390/diagnostics16162639

**Published:** 2026-08-19

**Authors:** Alina Florentina Pistrițu, Mihai Radu Pahomeanu, Andreea Irina Ghiță, Cosmin Mihail Andrian, Arina Ilinca Gheorghe, Elena Alupei, Dana Bilous, Deniz Gunsahin, Cătălina Vlăduț, Adrian Costache, Petruța Violeta Filip, Cristina Tocia, Răzvan Cătălin Popescu, Cristian George Țieranu, Eugen Dumitru, Mihai Ciocîrlan, Corina Silvia Pop, Lucian Negreanu

**Affiliations:** 1 Faculty of Medicine, “Carol Davila” University of Medicine and Pharmacy, 050474 Bucharest, Romaniapetruta.filip@umfcd.ro (P.V.F.); lucian.negreanu@umfcd.ro (L.N.); 2 Internal Medicine and Gastroenterology Department, University Emergency Hospital of Bucharest, 050098 Bucharest, Romania; 3 Gastroenterology Department, Emergency Clinical Hospital of Bucharest, 014461 Bucharest, Romania; 4 Gastroenterology Department, “Prof. Dr. Agrippa Ionescu” Hospital, 011356 Bucharest, Romania; 5 Faculty of Medicine, “Ovidius” University of Constanta, 900470 Constanta, Romania; cristina.tocia@yahoo.com (C.T.); eugen.dumitru@yahoo.com (E.D.); 6 Gastroenterology Department, “Sf. Apostol Andrei” Emergency County Hospital, 900591 Constanta, Romania; 7 Abdominal Surgery Department, “Sf. Apostol Andrei” Emergency County Hospital, 900591 Constanta, Romania; 8 Gastroenterology Department, Elias Emergency Clinical Hospital of Bucharest, 011461 Bucharest, Romania; 9 Research and Development Center for Morphological and Genetic Studies in Malignant Pathology (CEDMOG), “Ovidius” University of Constanta, 900470 Constanta, Romania; 10 Academy of Romanian Scientists, 050044 Bucharest, Romania

**Keywords:** artificial intelligence, large language model, chatbot, acute-on-chronic pancreatitis, pancreatitis

## Abstract

**Background/Objectives:** Acute-on-chronic pancreatitis (ACP) is a fibro-inflammatory syndrome that stands at the border between acute pancreatitis (AP) and chronic pancreatitis (CP). It can be broadly defined as an acute exacerbation of CP. Large language models (LLMs) are artificial intelligence (AI) machine learning tools that can be employed in clinical decision systems (CDSs). **Methods:** This is an exploratory benchmark study. Six LLM chatbots were evaluated on a Likert scale by a panel of 13 experts in pancreatology based on the similarity of responses to a position statement (PS) in ACP. **Results:** ClaudeAI had the best average Likert score, while Alice had the lowest. There was a positive correlation between the length of the response and expert-perceived similarity. There was no correlation between seniority and Likert score. **Conclusions:** In this exploratory benchmark, some LLMs generated answers that were often judged as similar to the position statement, suggesting that they might be helpful as educational support tools and may assist in the refinement of research questions or in the identification of research gaps in this particular pathology.

## 1. Introduction

Chronic pancreatitis (CP) is a fibro-inflammatory syndrome characterized by an ongoing inflammation of the pancreas [1]. An acute worsening of the inflammatory process already present in CP, causing a deterioration in a patient’s clinical condition, defines acute-on-chronic pancreatitis (ACP) [2]. Within the known literature, this condition might also be referred to as an acute exacerbation of CP or a flare-up of CP [3]. It is estimated that roughly 12–14% of the admissions for acute inflammation of the pancreas are in fact ACP [2]. ACP is typically associated with recurrent AP and is more prevalent among younger individuals than CP [4]. Despite the growing use of LLM-based tools in several medical fields, their performance regarding ACP in particular remains largely unexplored.

Chatbots are artificial intelligence software applications meant to replicate human conversation, whether through text or through speech. It was shown that these chatbots have the capacity to mimic homophily [5]. Researchers have been increasingly interested in the ways these chatbots, as well as other sorts of artificial intelligence (AI), can interact with current healthcare models, both in beneficial ways and otherwise [6]. LLMs and their implementation in this domain have been previously studied, from evaluating their feasibility in creating healthcare assistants for telemedicine purposes [7] to evaluating their possible role in clinical practice.

While there have been other studies regarding specific models in pancreatology [8] at the date of writing this article, we were not able to find any benchmark study for multiple widely available LLM chatbots. Considering the growing importance of this type of AI algorithm in helping medical students in training, junior medical staff in their duties, and clinical researchers in defining aims for their future studies, we sought to find out how different LLM chatbots compare in terms of similarity to a reference framework. While their performance has been explored across several medical domains, their reliability in highly complex and weakly standardized conditions remains largely unknown. ACP therefore represents a stringent stress test for the clinical fidelity of LLM-generated medical advice.

As such this study aims to compare multiple chatbot responses, evaluated by a team of experts in pancreatology with respect to a position statement published in this field [2]. The primary aim of the research was, thus, to explore whether the studied LLMs gave similar answers to the PS. Secondary aims were to compare the perceived similarity of chatbot responses in regard to the length of their responses, while evaluating the seniority level of the experts in regard to the Likert scores and checking to see if there was any type of correlation between expert seniority level and their scoring behavior.

In this paper, the Materials and Methods section describes the study design, the Results section presents the main findings and the Discussion section deals with the implications and limitations of this study.

## 2. Materials and Methods

No patient data were involved in this study. This study was cleared for ethical standards by the Ethical Committee of University Emergency Hospital of Bucharest (no. of approval: 68629/20; date: 18 October 2024). For the Discussion section, Scite (Scite Inc., New York, NY, USA) was used to help refine and find some of the references. All the references provided by Scite were reviewed by one of the authors (M.R.P.) and there are no parts of this paper that were written by AI tools.

This was an exploratory single-blinded neutral benchmark study for which we took into consideration all of the available LLM chatbots on 2 October 2024, as stated on Wikipedia (Wikimedia Foundation Inc., San Francisco, CA, USA) (see Supplementary Files). Exclusion criteria used for chatbots: inactive website, unavailable on Windows Home 11 (Microsoft Corporation, Redmond, WA, USA), designed for non-medical usage, protected by/restricted behind paywall, lack of availability in Romania for large audiences, and provided repeated answers unrelated to the questions. These exclusion criteria were used as this study sought public accessibility for reproducibility and not technical superiority. All the chatbots were interrogated in the official language of the country where they had their headquarters. For the chatbot located outside the USA, we used Google Translate (Alphabet Inc., Mountain View, CA, USA) to translate the questions and answers from English to Russian and backwards. Details are shown in Table 1.

In order to suppress any potential history of interaction between the inquirers and the chatbots we opened accounts at all the inquired chatbots with a new e-mail address. All the inquirers were instructed to use Google Chrome v130 (Alphabet Inc., Mountain View, CA, USA) and to clear their browsing history before questioning the chatbots. The inquirers had to input into the chatbot the exact question that was found in the position statement (PS) [2] previously published by Bouça-Machado et al. regarding ACP. The exact LLM versions were approximated based on the best publicly available records. See Table 1 for details. All the inquiry statements were based on the exact questions raised by the PS.

Medical doctors trained or in training to become gastroenterologists, internists or general (abdominal) surgeons, who had a clinical experience of at least three years in the field of pancreatology and held a PhD diploma or were PhD candidates, were recruited to the panel of experts. All the experts were board-certified physicians and proficient in the English language. A total of 13 experts were recruited from the following two universities: Carol Davila University of Medicine and Pharmacy (Bucharest, Romania, EU) and Ovidius University (Constanța, Romania, EU). Throughout the evaluation process, the experts were blinded regarding the identity of the evaluated chatbots by using a standardized expert questionnaire (EQ) in order to minimize brand or reputation bias in their ratings. See the Supplementary Files for a model of the EQ used in this process. In the EQ the experts were required to declare their number of years of clinical experience in pancreatology and to assess the similarity of chatbots’ responses in comparison to the responses found in the PS [2].

The assessment was standardized by Likert scale [9] and all 5 values on the scale were associated with a statement regarding the similarity of the response, as shown in Table 2. The expert’s Hirsch index [10] was obtained from public records available from Google Scholar (Alphabet Inc., Mountain View, CA, USA) on 1 February 2025. See the Supplementary Files.

The EQ was completed by the experts using Microsoft Office 365 (Microsoft Corporation, Redmond, WA, USA); the results were organized with Google Docs (Alphabet Inc., Mountain View, CA, USA) and Microsoft Office 365. All the statistical analyses required in this study were run with SPSS v30.0.0.0 (IBM Corp., Armonk, NY, USA). The statistical tests used in this study were means, Kruskal–Wallis with Pairwise Comparison for post hoc analysis, and Pearson correlation, while for assessing interrater agreement Intraclass Correlation Coefficient (ICC) was used. The ICC was calculated based on the average Likert scores for all questions across the six chatbots which were rated by 13 experts, with a total of 780 observations. A statistically significant result was reported only when the two-tailed *p*-value was under 0.05 or when the confidence interval (CI 95%) excluded 1.00. Zotero v7.0.11 (Corporation for Digital Scholarship, Vienna, VA, USA) and its extensions were used to manage references.

This study is an exploratory, expert-based benchmark and does not constitute a formal clinical validation of ACP management recommendations.

## 3. Results

### 3.1. Chatbots Included in the Study

**Figure 1:**
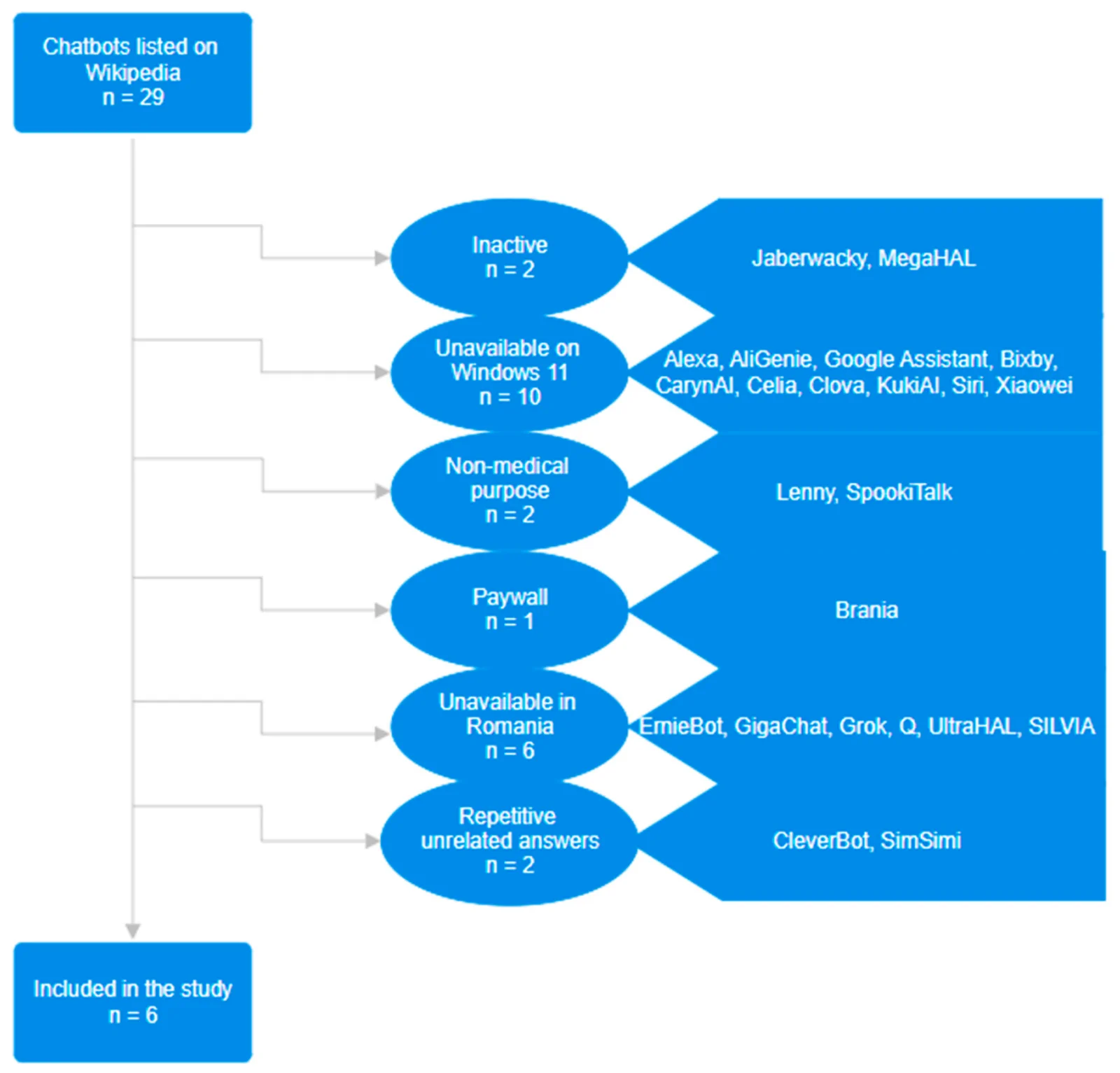
Flowchart of chatbots included in the study.

From the 29 chatbots listed on Wikipedia, after applying the exclusion criteria, we included 6 chatbots in this study. See Figure 1 for the flowchart. Most of the chatbots were excluded for their lack of availability on Windows (34.48%) and for their lack of availability for the general audience in Romania (20.69%). A comprehensive list of all the chatbots listed on Wikipedia on 2 October 2024 can be found in the Supplementary Files.

### 3.2. Comparing the Performance of the Chatbots

In order to compare the performance of chatbots, we used a Kruskal–Wallis test that indicated a significant difference in Likert scores across the six chatbots examined: χ^2^(5, *N* = 780) = 108.08, *p* < 0.01. The median rank of the Likert score was 4.00 for ChatGPT, ClaudeAI and Gemini; 3.00 for Brave Leo and Copilot; and 2.00 for Alice.

Post hoc comparisons using Pairwise Comparisons indicated that the median-rank Likert score of Alice was significantly lower than that of all other chatbots; see the Supplementary Files for *p*-values. Brave Leo had a significantly lower median rank than Copilot, *p* = 0.02; Gemini, *p* < 0.01; ChatGPT, *p* < 0.01; and ClaudeAI, *p* < 0.01. Copilot had a significantly lower median rank than ChatGPT, *p* < 0.01, and ClaudeAI, *p* < 0.01, and Gemini had a significantly lower median rank than ClaudeAI, *p* < 0.01. However, there was no significant difference between the median-rank Likert score of ChatGPT and ClaudeAI, *p* = 0.58; Copilot and Gemini, *p* = 0.28; and ChatGPT and Gemini, *p* = 0.10.

For this aim, as the medians were so close and there were still statistical differences seen in the Kruskal–Wallis test, we decided to also calculate the mean Likert score. ClaudeAI had the highest mean Likert score (M = 3.79, SD = 0.89) and Alice the lowest (M = 2.44, SD = 1.21). See Table 3 for details.

### 3.3. Comparison of Alignment with PS Regarding the Length of the Response

For the comparison of the performance of chatbots in regard to the length of their responses, we compared the means of their Likert scores with the means of their lengths of response. See Table 3 and Table 4 for details.

**Figure 2:**
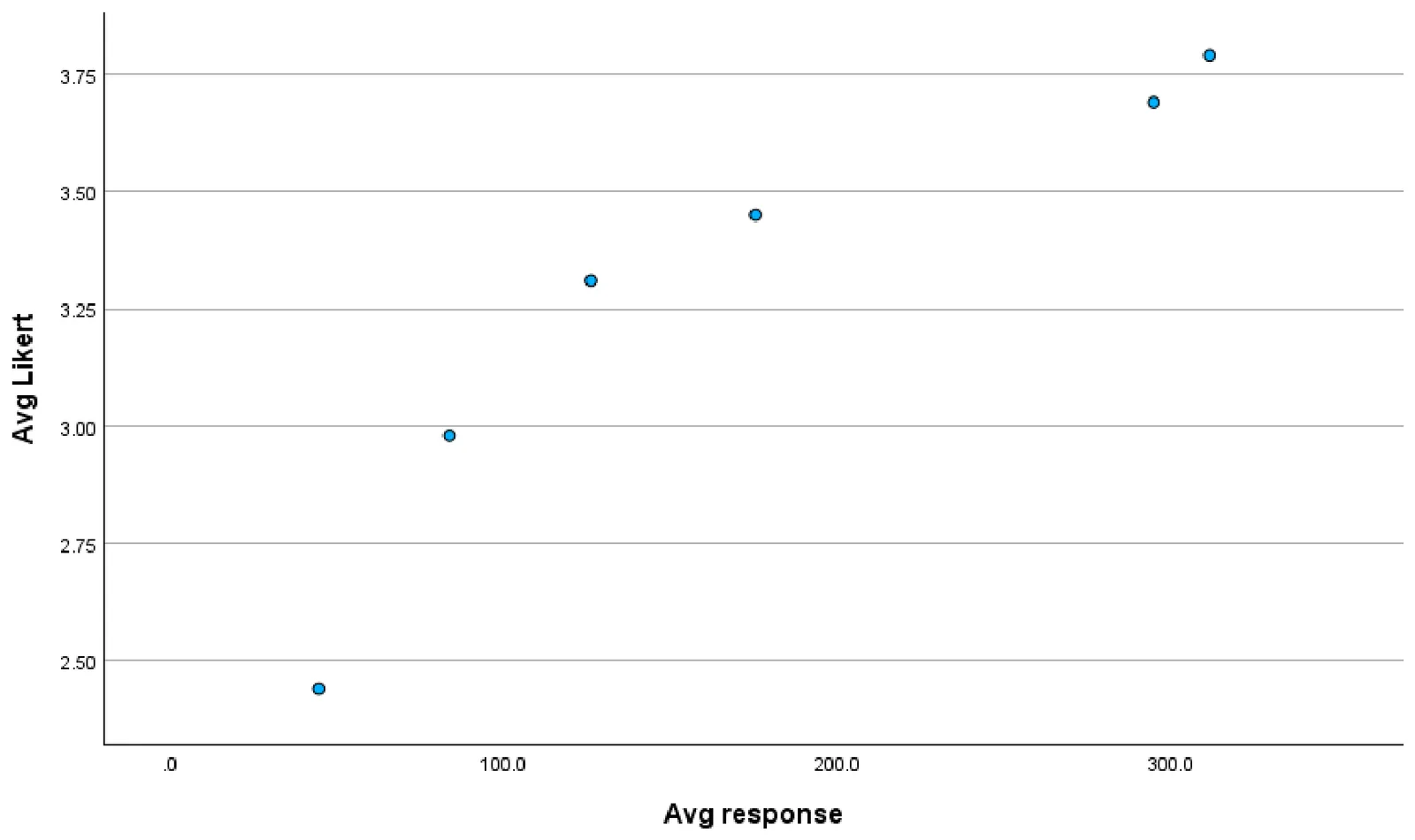
Average length of response in regard to the average Likert score.

A Pearson correlation coefficient was calculated to assess the linear relationship between the average Likert score and the mean length of response. There was a positive correlation between the two variables r(4) = +0.92, *p* < 0.01. See Figure 2.

### 3.4. Evaluation of Seniority Level of the Experts Regarding Their Likert Score

To assess the seniority level of the experts, we took into consideration the Hirsch index and self-declared years of experience in the field of pancreatology. See the Supplementary Files for details. The highest Hirsch index was that of L.N. (h-index = 21) while the lowest was that of A.F.P. (h-index = 0). The highest experience in practice was that of C.S.P. and R.C.P. (years = 25), while the lowest was that of D.B. and D.G. (years = 3). See the Supplementary Files for details.

**Figure 3:**
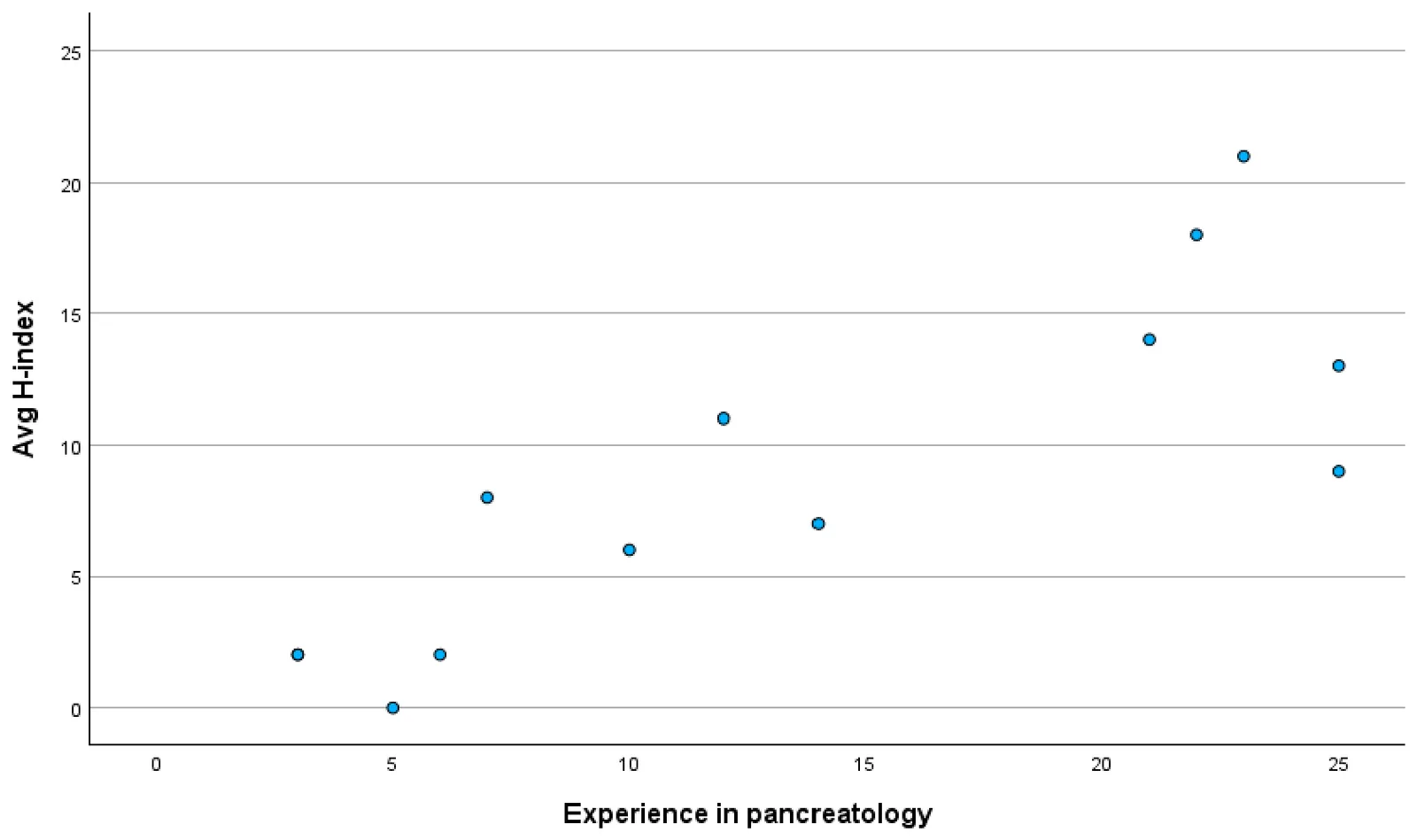
Experience in pancreatology (years) in regard to Hirsch index.

Pearson correlation coefficients were calculated to assess the linear relationships between the experts’ seniority measures and their average Likert scores. There was a positive correlation between experience and the Hirsch index, r(11) = +0.84, *p* < 0.01. Meanwhile, we observed no statistically significant correlation between the median Likert score and either the Hirsch index, r(11) = +0.04, *p* = 0.91, or experience, r(11) = +0.24, *p* = 0.43. See Figure 3.

Interrater agreement was assessed based on the Intraclass Correlation Coefficient (ICC) [11]. The ICC was calculated using a two-way mixed-effects model to assess the consistency of average measures across raters. The ICC value was 0.96 (95% CI [0.90, 0.99]), indicating excellent reliability. This value suggests good agreement among raters when evaluating a chatbot’s performance.

## 4. Discussion

In line with the rapid expansion of AI-based clinical decision support systems in medicine, our benchmark illustrates that formal, domain-specific validation of LLM tools in pancreatology remains limited. In this benchmark, none of the evaluated LLMs achieved complete concordance with the position statement on acute-on-chronic pancreatitis, and there were statistically significant differences observed. Expert seniority was not associated with differences in Likert-based scoring, suggesting that evaluations of LLM-generated responses were consistent across experience levels. Moreover, interrater agreement showed excellent reliability, indicating a high level of consistency among expert ratings.

LLMs, as a particular subtype of AI machine learning, are being widely used in medicine [12]. Their main applications are summarizing the previous literature and summarizing electronic health records. These applications and their ease of use make them one of the most used AI tools in medicine, mainly by junior doctors and medical students. LLMs now hold great potential in transforming clinical practice by improving medical learning, patient communication, and clinical decision-making [13]. As they are a relatively new tool in practice, there is a higher demand to evaluate their performance.

The usage of AI solutions in medicine has gained impressive traction in the past few years in both medicine and clinical research. AI tools are well embraced by medical students, with more than 85.0% of them showing enthusiasm in learning more about their applications in medicine [14] and 89.8% considering using them in their practice in the next 10 years, but less than 2.5% consider them useful in gastroenterology [15]. LLMs were previously [16] found to be helpful in assisting junior doctors’ decisions. While there is growing usage of LLMs in academic writing, as shown by previous studies [17], in medical research there are growing concerns regarding not only ethical integrity but also LLM accuracy [18]. With perceived improvements in AI tools, Clinical Decision Support systems (CDSs) have outgrown the possibility of timely clinical validation [19,20]. The practice of using AI-assisted tools in medicine was augmented during recent public healthcare crises, such as the COVID-19 pandemic [21]. In gastroenterology, there are several CDSs supported by AI tools that have found some favorable reception from clinicians [22], but most of them are useful in gastroenterological endoscopy [23,24]. Meanwhile, none of the LLM tools largely available are validated now for clinical management, as most of them still have issues with hallucinations [25] and they still cannot replace imaging, labs, guidelines or clinical judgment.

Pancreatology, being a narrower field of clinical medicine, has fewer studies regarding validation of AI tools used for CDSs. In this field, AI solutions are used mostly in imaging techniques deployed for pancreatic neoplasia detection, with a systematic review by Podina et al. finding 95 recent studies on this particular topic. While some showed unprecedented diagnostic accuracy, none of the AI solutions investigated were using LLMs [26]. A recent narrative review by Nastac et al. [27] exploring the AI tools used in pancreatology found no studies regarding the use of LLMs in acute or chronic pancreatitis. In acute and chronic pancreatitis, Nastac et al. emphasized that artificial neural networks might be reliable in identifying autoimmune pancreatitis.

The worst results in this study came from a chatbot designed to run in another language, as the PS was written in English. A third party was used (i.e., Google Translate) to translate the questions back and forth. This could have been a limitation of our study regarding Alice, as it underperformed compared with its counterparts. Another limitation could derive from using a PS paper [2] and not a guideline, as there are no guidelines available regarding ACP to date. Meanwhile, a bibliometric study found that a significant part of the gastroenterological guidelines are based on low-quality evidence [28]. As some could consider inviting young experts to the panel a limitation, this study aimed to also evaluate if seniority could somehow affect the ratings that the experts gave and as such it was important for it to also have young experts in the panel. Nevertheless, it seems that seniority and experience did not play a role in evaluating LLM chatbots and the ICC proved to have good agreement among experts in this study.

This study has several limitations, as the expert ratings reflect perceived similarity to PS, not direct factual verification, and ACP to date remains a heterogeneous and consensus-based pathology. As there is no universally validated scoring system for ACP-related chatbot outputs, we used a standardized Likert scale anchored in expert judgment. It should be acknowledged that the use of automated translation for the Russian-language chatbot may have introduced semantic distortions, potentially underestimating its performance. Repeated prompting could have provided different answers and was outside of the scope of this study, as the limit of repeating the same question is unclear and could positively influence the chatbot, but this could be interpreted by some as a limitation of this study. The exclusion of subscriber-based LLM versions could affect the results of this study. No exact identifiers such as temperature settings or API tags were taken into account for this study. It is worth mentioning that LLMs evolve rapidly, so the results might not reflect the versions available when this study is published. Future studies should compare zero-shot, few-shot, chain-of-thought, structured, retrieval-augmented, and guideline-assisted prompting to determine whether optimized prompting strategies improve ACP-related responses beyond the baseline performance reported here. Consequently, the present results should be viewed as exploratory and hypothesis-generating rather than as definitive validation of LLM suitability for clinical decision support.

In this exploratory benchmark, some LLMs generated responses that were judged to be similar to the chosen framework, suggesting potential educational and decision support roles in ACP. For them to be included in clinical practice, there is still room for improvement. Newer versions of LLMs tend to have substantially improved accuracy in the broader pancreatology field [29].

Future work on this particular topic could use scenario-based vignettes, test newer model generations, include multilingual evaluations with professional translations, and, when a guideline is available, could evaluate factual correctness versus guideline recommendations, if not for this particular pathology, then for other better defined and more frequently encountered entities in pancreatology. Çamur et al. [30] already showed that hyperparameter optimization and structured input design significantly improve diagnostic accuracy and clinical usefulness of LLMs. Studying any clinical consequence like clinical danger stratification of the responses and domain mapping could prove to offer valuable insights in particular for clinicians if they are made against a widely embraced guideline.

## 5. Conclusions

In this exploratory benchmarking study, we assessed the performance of multiple LLMs in relation to a PS previously published about ACP using expert rating.

Some LLMs achieved higher expert-rated similarity scores; ClaudeAI marginally outperformed the rest in mean similarity to the PS, as evaluated by expert ratings. Length of the response correlated positively with a higher perceived similarity. Seniority did not affect the scoring of the experts involved in this study.

This exploratory study indicates that LLMs might be helpful in optimal conditions for medical research. However, this study was not designed to demonstrate that LLMs are suitable as standalone tools for ACP management.

## Figures and Tables

**Table 1:** List of LLM chatbots included in the study (ordered alphabetically).

Chatbot	Website	Company	Native Language	Model Version
Alice	https://alice.yandex.ru/	Yandex (Moscow, Russian Federation)	Russian	YandexGPT 3
Brave Leo	https://search.brave.com/	Brave Software Inc. (San Francisco, CA, USA)	English	Mixtral 8 × 7B
ChatGPT	https://chatgpt.com/	OpenAI (San Francisco, CA, USA)	English	GPT-3.5
ClaudeAI	https://claude.ai/	Anthropic (San Francisco, CA, USA)	English	Claude 3 (Opus)
Copilot	https://copilot.microsoft.com/	Microsoft Corporation (Redmond, WA, USA)	English	Not publicly disclosed
Gemini	https://gemini.google.com/app	Alphabet Inc. (Mountain View, CA, USA)	English	Gemini 1.5 (Flash)

**Table 2:** Likert scale used in this study.

Likert Scale	Statement
5	Identical to PS
4	Very similar to PS
3	Moderately similar to PS
2	Slightly similar to PS
1	No similarity to PS

**Table 3:** Mean Likert score obtained by chatbots.

Chatbot	Mean Likert Score	Standard Deviation
ClaudeAI	3.79	0.89
ChatGPT	3.69	1.08
Gemini	3.45	1.17
Copilot	3.31	1.11
Brave Leo	2.98	1.20
Alice	2.44	1.21

**Table 4:** Length of response by chatbot.

Chatbot	Mean Length of Response (Words)
ClaudeAI	311.50
ChatGPT	294.80
Gemini	175.50
Copilot	126.50
Brave Leo	83.80
Alice	44.70

## Data Availability

The original contributions presented in this study are included in the article/Supplementary Materials. Further inquiries can be directed to the corresponding author.

## Supplementary Materials

The following supporting information can be downloaded at https://www.mdpi.com/article/10.3390/diagnostics16162639/s1, Supplementary File S1—Seniority; Supplementary File S2—Expert questionnaire; Supplementary File S3—Length of response; Supplementary File S4—Seniority; Supplementary File S5—Expert scores; Supplementary File S6—Statistical test Aim 1; Supplementary File S7—Statistical test Aim 2; Supplementary File S8—Statistical test Aim 3–4; Supplementary File S9—Wikipedia 2 Oct.
