# Supplementary material for: Benchmarking Large Language Models in Acute-on-Chronic Pancreatitis: An Exploratory Study
Source: Diagnostics (Basel). 2026 Aug 19;16(16):2639. doi: 10.3390/diagnostics16162639 (PMC13512442; doi:10.3390/diagnostics16162639)
Supplement: Supplementary file 1 [file diagnostics-16-02639-s001.zip › Supplementary File S1 - Seniority.pdf]

Expert	Hirsch index
Lucian Negreanu	21
Mihai Ciocîrlan	18
Eugen Dumitru	14
Corina Silvia Pop	13
Cristian Țieranu	11
Răzvan Cătălin Popescu	9
Cristina Tocia	8
Petruța Violeta Filip	7
Cătălina Vlăduț	6
Mihai Radu Pahomeanu	2
Deniz Gunsahin	2
Dana Bilous	2
Alina Florentina Pistrițu	0

Table S1.1 – Hirsch index of the experts involved in the study

Expert	Experience in pancreatology (years)
Corina Silvia Pop	25
Răzvan Cătălin Popescu	25
Lucian Negreanu	23
Mihai Ciocîrlan	22
Eugen Dumitru	21
Petruța Violeta Filip	14
Cristian Țieranu	12
Cătălina Vlăduț	10
Cristina Tocia	7
Mihai Radu Pahomeanu	6
Alina Florentina Pistrițu	5
Deniz Gunsahin	3
Dana Bilous	3

Table S1.2 -Self-declared experience of the experts involved in the study
