# Supplementary material for: Benchmarking Large Language Models in Acute-on-Chronic Pancreatitis: An Exploratory Study
Source: Diagnostics (Basel). 2026 Aug 19;16(16):2639. doi: 10.3390/diagnostics16162639 (PMC13512442; doi:10.3390/diagnostics16162639)
Supplement: Supplementary file 1 [file diagnostics-16-02639-s001.zip › Supplementary File S2 - Expert Questionnare.pdf]

Dear, [expert name], in the name of Romanian Acute Pancreatitis Index (RO-API) Study Group, formerly known as Bucharest Acute Pancreatitis Index (BUC-API), I would like to thank you for participating in the LLM-ACP Study. In this document you will find all the questions that are to be reviewed by you together with extracts from the position statement (PS) (https://www.sciencedirect.com/science/article/pii/S1424390323000352?via%3Dihub) to be easier for you to find them. Before starting, please complete the two fields in the Expert Data subsection. You will find at the beginning of this document also the Likert score legend. Rough estimate of time needed to complete the questionnaire: 1h 15 min. All the exact answers from the chatbots will be written in *italics*.

Questions will be codified with 4 digits. The first two will refer to the chatbot that was enquired, while the last two will refer to the question in the order that they were stated in the position paper.

As this is a single-blinded study, if you are interested, we are happy to provide you, at request, exact information regarding the chatbots inquired prior to drafting the manuscript but after your EQ is finished. In the end I would like to thank you for your keen interest in pancreatology research and especially RO-API projects.

In the supplementary files of the study we will include only a general model of this EQ. If there are any questions left, feel free to contact us at: mihairadu.pahomeanu@gmail.com or +40724039210.

Deadline for completion of EQ: 1st December 2024.

On behalf of RO-API Study Group,

Mihai Radu Pahomeanu & Prof. Lucian Negreanu

Carol Davila University of Medicine and Pharmacy

University Emergency Hospital of Bucharest

EXPERT DATA

Expert unique code: XXX

e-mail:____________________________

Telephone:__________________________

Clinical experience in treating AP: _____ (years)

Rough estimate of number of AP treated to date: ______

LIKERT SCORE LEGEND

Likert score	Meaning
1	No similarity to PS
2	Slightly similar to PS
3	Moderately similar to PS
4	Very similar to PS-ACP
5	Identical to PS-ACP

<u>QXX01: What is the best term to describe an acute pancreatic inflammation in chronic pancreatitis?</u>

Extract from the position statement:

Question 1: What is the best term to describe an acute pancreatic inflammation in CP?

Answer: "Acute on chronic pancreatitis" is the best term to describe an acute pancreatic inflammation in patient with CP.
Quality assessment: Low
Recommendation: Conditional
Agreement: Strong

Commentary: In the literature, several terms are used to describe an acute pancreatic inflammation in a patient with CP. The two most frequently used terms are "acute on chronic pancreatitis" and "(acute) exacerbations of chronic pancreatitis" [10]. The terms "exacerbation of chronic pancreatitis" and "acute exacerbation of chronic pancreatitis" are ambiguous as it is not clear whether this is referring to increased clinical symptoms (e.g., pain aggravation in setting of a chronic pain syndrome), altered biochemistry (e.g., elevated serum pancreatic enzyme levels), new radiological features (e.g., peri- and pancreatic oedema, acute fluid collection and/or inflammatory mass) or a combination of these. The same issues apply to the term "acute on chronic pancreatitis" which also suggests AP superimposed on CP. This is not strictly accurate since a second disease process (AP) is unlikely, but the condition rather represents an acute worsening in the already present inflammation associated with CP. It is worth noting that the phrase "acute on chronic" is often used in other disease settings, including liver failure ("acute-on-chronic liver failure") and kidney disease ("acute-on-chronic kidney disease"), and is meant to imply an acute worsening of a chronic disease [22–26]. Because "acute on chronic pancreatitis" is used most often in the literature [10] and is probably better understood, there was consensus by the EP that this is the preferred term. When new causes for an acute pancreatic inflammation occurs in a patient with CP as e.g., after endoscopic retrograde cholangiopancreatography (ERCP), this should normally be labelled post-ERCP AP rather than ACP.

Q0101: What is the best term to describe an acute pancreatic inflammation in chronic pancreatitis?

Chatbot answer: *Acute inflammation of the pancreas in chronic pancreatitis is called acute pancreatitis.*

LIKERT SCORE = _______

Q0201: What is the best term to describe an acute pancreatic inflammation in chronic pancreatitis?

Chatbot answer: *The best term to describe acute pancreatic inflammation that occurs in the context of chronic pancreatitis is "acute-on-chronic pancreatitis."*

This term indicates a situation where a patient with a history of chronic pancreatitis experiences an acute exacerbation or inflammatory episode. This condition can lead to increased pain and complications and requires careful management, often involving both medical and surgical interventions.

LIKERT SCORE = ______

Q0301: What is the best term to describe an acute pancreatic inflammation in chronic pancreatitis?

Chatbot answer: *The best term to describe an acute pancreatic inflammation occurring in the setting of chronic pancreatitis is "acute exacerbation of chronic pancreatitis" or "acute-on-chronic pancreatitis."*

This term accurately conveys that there is an acute inflammatory episode superimposed on an underlying chronic condition of the pancreas. It distinguishes the event from a standalone acute pancreatitis episode in a previously healthy pancreas.

Would you like more information about the characteristics or management of acute-on-chronic pancreatitis?

LIKERT SCORE = ______

Q0501: What is the best term to describe an acute pancreatic inflammation in chronic pancreatitis?

Chatbot answer: *The best term to describe an acute pancreatic inflammation occurring in the context of chronic pancreatitis is acute exacerbation of chronic pancreatitis. This term indicates a sudden worsening of inflammation in a pancreas that is already chronically inflamed.*

Is there anything specific you would like to know about this condition?

LIKERT SCORE = ______

Q0601: What is the best term to describe an acute pancreatic inflammation in chronic pancreatitis?

Chatbot answer: *Acute exacerbation of chronic pancreatitis is the most accurate term to describe an acute inflammatory flare-up in a patient with pre-existing chronic pancreatitis.*

This condition is characterized by a sudden worsening of symptoms, often including severe abdominal pain, nausea, vomiting, and fever. It can be a serious complication of chronic pancreatitis and may require hospitalization.

LIKERT SCORE = ______

Q0701: What is the best term to describe an acute pancreatic inflammation in chronic pancreatitis?

Chatbot answer: *In chronic pancreatitis, an acute inflammation of the pancreas is still referred to as acute pancreatitis. The terms don't change based on the chronicity of the condition. However, repeated episodes of acute pancreatitis can contribute to the development of chronic pancreatitis.*

LIKERT SCORE = ______

QXX02: What is the optimal definition of acute on chronic pancreatitis?

Extract from the position statement:

Question 2: What is the optimal definition of ACP?
Answer: ACP is defined as "an acute worsening of the inflammatory process associated with CP, resulting in a deterioration of the patient's clinical condition, typically resulting in increased pancreatic pain" (Fig. 2).
Quality assessment: Low

Recommendation: Conditional
Agreement: Strong

Commentary: There are several definitions of ACP in the literature [10]. A good definition should be easily understood and include the fundamental features that characterize ACP, or more specifically distinguish ACP from AP and CP. These features are the presence of co-existent CP, the acute onset, the increased inflammation and a deterioration in the patient's clinical condition. This definition is an adaption of the definition from Olesen et al. [27] that was the most comprehensive and meaningful definition found in the previous literature review [10]. CP is characterized by an ongoing fibroinflammatory process and, as such, inflammation is already present in many patients [1]. Therefore, ACP is most often considered an increase in the underlying pancreatic inflammation and not a *de novo* inflammation. The recommendation to use the phrase "patient's clinical condition" (rather than specifically refer to increased abdominal or pancreatic pain) is because patients can present with a range of variable symptoms and/or signs, including jaundice and duodenal obstruction [28–31]. ACP is distinguished from recurrent acute pancreatitis by the diagnosis of CP prior to ACP. The authors do, however, appreciate this can be difficult in the clinical setting.

Q0102: What is the optimal definition of acute on chronic pancreatitis?

Chatbot answer: *The optimal definition of acute pancreatitis is acute inflammation, not associated with infection, that leads to necrosis of pancreatic cells and their damage. The optimal definition of chronic pancreatitis is long-term inflammation of the pancreas, in which the structure of the tissue and ducts is irreversibly changed, which is manifested by pain and/or persistent deterioration in the functioning of the organ.*

LIKERT SCORE = ______

Q0202: What is the optimal definition of acute on chronic pancreatitis?

Chatbot answer: *Acute-on-chronic pancreatitis is defined as the occurrence of an acute inflammatory episode in a patient with established chronic pancreatitis. This condition is characterized by a sudden exacerbation of symptoms, such as severe abdominal pain, nausea, vomiting, and potential complications like pancreatic necrosis or infection, in the backdrop of underlying chronic pancreatic damage.*

Key Features of Acute-on-Chronic Pancreatitis:

1. Underlying Chronic Condition: Patients have a history of chronic pancreatitis, which may be due to factors like chronic alcohol use, genetic predispositions, or other etiologies.

2. Acute Inflammatory Episode: There is a new onset of acute inflammation, which can lead to increased pain and may present with typical symptoms of acute pancreatitis.

3. Complications: The acute episode may result in complications such as:

o Pancreatic necrosis

o Infected pancreatic collections

o Pseudocyst formation

o Biliary obstruction

4. Diagnostic Criteria: The diagnosis typically involves imaging studies (like CT or MRI) and laboratory tests (such as elevated pancreatic enzymes).

5. Management: Treatment may require hospitalization, supportive care, pain management, and sometimes surgical intervention, depending on the severity and complications.

Clinical Importance:

Understanding acute-on-chronic pancreatitis is crucial for appropriate management and intervention, as it can significantly affect patient outcomes

LIKERT SCORE = ______

Q0302: What is the optimal definition of acute on chronic pancreatitis?

Chatbot answer: *The optimal definition of acute-on-chronic pancreatitis combines elements from both acute and chronic pancreatitis, while recognizing the unique aspects of this clinical entity. Here's a comprehensive definition:*

Acute-on-chronic pancreatitis is defined as an acute exacerbation of inflammation in a patient with pre-existing chronic pancreatitis, characterized by:

Sudden onset or worsening of abdominal pain typical of pancreatitis

Elevation of pancreatic enzymes (amylase and/or lipase) to at least three times the upper limit of normal

Evidence of acute pancreatic inflammation on imaging studies (such as CT or MRI)

Occurring against the background of established chronic pancreatitis, as evidenced by:

a. Known history of chronic pancreatitis, or

b. Imaging findings consistent with chronic pancreatic changes (e.g., calcifications, ductal abnormalities, parenchymal atrophy)

Potential for complications distinct from those seen in isolated acute or chronic pancreatitis

This definition incorporates clinical symptoms, laboratory findings, imaging criteria, and the context of pre-existing chronic disease. It also acknowledges that acute-on-chronic pancreatitis may have unique features or complications that differ from either acute or chronic pancreatitis alone.

It's worth noting that there isn't a universally accepted definition, and ongoing research may refine this further. Would you like to discuss any specific aspects of this definition or how it's applied in clinical practice?

LIKERT SCORE = ______

Q0502: What is the optimal definition of acute on chronic pancreatitis?

Chatbot answer: *Acute on chronic pancreatitis refers to an acute exacerbation of inflammation in a pancreas that is already affected by chronic pancreatitis. This condition represents a worsening of the chronic inflammatory process, leading to a deterioration in the patient's clinical condition and increased pancreatic pain1. Essentially, it combines features of both acute and chronic pancreatitis, highlighting the continuum between these two states rather than treating them as entirely separate conditions2.*

Would you like more information on the symptoms or management of this condition?

LIKERT SCORE = ______

Q0602: What is the optimal definition of acute on chronic pancreatitis?

Chatbot answer: *Acute on Chronic Pancreatitis: A Complex Definition*

Acute on chronic pancreatitis (ACP) is a complex condition characterized by a sudden exacerbation of symptoms in a patient with pre-existing chronic pancreatitis. It's a challenging diagnosis to make, as it often presents with similar symptoms to acute pancreatitis but in the context of a chronic condition.

A widely accepted definition is:

- *An acute worsening of the inflammatory process associated with chronic pancreatitis.*

- *Typically resulting in a deterioration of the patient's clinical condition, often manifested as increased pancreatic pain.*

Key features to consider:

- *History of chronic pancreatitis: A pre-existing diagnosis of chronic pancreatitis is essential.*
- *Acute exacerbation: A sudden worsening of symptoms, often characterized by increased pain, nausea, vomiting, or other gastrointestinal distress.*
- *Laboratory and imaging findings: Elevated pancreatic enzymes and imaging studies (e.g., CT, MRI) may show evidence of acute inflammation or worsening of chronic changes.*

It's important to note:

- *The differentiation between acute pancreatitis and ACP can be challenging.*
- *The underlying causes of ACP may vary and can include factors like alcohol abuse, gallstones, or medication side effects.*
- *Management strategies often involve a combination of pain control, supportive care, and addressing any underlying causes.*

If you have concerns about your or someone else's health, please consult with a healthcare professional. They can provide a proper diagnosis and recommend appropriate treatment.

LIKERT SCORE = ______

Q0702: What is the optimal definition of acute on chronic pancreatitis?

Chatbot answer: *"Acute on chronic pancreatitis" refers to a situation where a patient with underlying chronic pancreatitis experiences an acute episode of pancreatitis. It's like having a chronic illness and then having an acute flare-up or worsening of symptoms. This can lead to further damage and complications in the pancreas.*

LIKERT SCORE = ______

QXX03: What is the incidence of acute on chronic pancreatitis?

Extract from the position statement:

Question 3: What is the incidence of ACP?

Answer: The incidence of ACP is difficult to accurately determine because of the lack of a consistent definition in the literature.

Quality assessment: Very low

Recommendation: Conditional

Agreement: Strong

Commentary: There are no reliable data to determine the incidence of ACP. This stems from the lack of a universally accepted definition of ACP. The literature indicates an increase in the diagnosis of ACP during emergency department visits [2] and the incidence of ACP appears to be increasing relative to AP [33]. This might reflect a true increase in the incidence of ACP or alternatively an increased awareness of ACP, and improved diagnostic accuracy due to the more widespread use of cross-sectional imaging modalities. About 12–14% of the admissions for acute pancreatic inflammation are in patients with CP [2,33,34]. A small cohort study of patients with alcohol related CP found an average of 2 (and a maximum of 5) episodes of ACP over a two-year period [35]. In contrast, in a retrospective study with the duration of 20 years, of 1415 patients with CP only 3.5% were diagnosed with ACP [36]. Given that there was no definition or diagnostic criteria for ACP in these two studies, it is difficult to draw any meaningful incidence estimate from these conflicting results.

Q0103: What is the incidence of acute on chronic pancreatitis?

Chatbot answer: *Over the past 30 years, the prevalence of acute and chronic pancreatitis worldwide has more than doubled. In Russia, chronic pancreatitis is diagnosed in 27–50 people per 100,000 population.*

LIKERT SCORE = ______

Q0203: What is the incidence of acute on chronic pancreatitis?

Chatbot answer: *The incidence of acute-on-chronic pancreatitis can vary widely based on several factors, including the population studied, underlying causes of chronic pancreatitis, and geographical location. However, some general statistics can provide an overview:*

1. General Incidence:

o Studies suggest that the incidence of acute-on-chronic pancreatitis can range from 20% to 30% in patients with chronic pancreatitis, particularly in those with a history of alcohol use.

2. Increased Risk in Specific Populations:

o Patients with chronic pancreatitis due to alcohol consumption are more likely to experience acute exacerbations. The risk increases with the duration and severity of alcohol use.

o Other underlying conditions, such as hereditary pancreatitis or autoimmune pancreatitis, may also influence incidence rates.

3. Hospitalization Rates:

o Acute exacerbations of chronic pancreatitis are a common reason for hospitalization. It is estimated that 5% to 10% of all hospital admissions for pancreatitis may be attributed to acute-on-chronic episodes.

4. Long-term Complications:

o Patients with chronic pancreatitis have a cumulative risk of experiencing acute exacerbations over time. Studies indicate that the risk of acute-on-chronic pancreatitis episodes can increase significantly after 10–15 years of living with chronic pancreatitis.

Summary

While precise incidence rates may vary, acute-on-chronic pancreatitis is a significant concern in patients with chronic pancreatitis, particularly in those with identifiable risk factors like alcohol use. Continued research and better understanding of the condition will help in managing and preventing these acute episodes effectively.

LIKERT SCORE = ______

Q0303: What is the incidence of acute on chronic pancreatitis?

Chatbot answer: *The incidence of acute-on-chronic pancreatitis is not as well-established as the incidence of acute or chronic pancreatitis individually. This is partly due to variations in definition, diagnostic criteria, and reporting practices. However, I can provide some insights based on available data:*

Estimated range: Studies suggest that acute-on-chronic pancreatitis occurs in approximately 16-23% of patients with known chronic pancreatitis.

Variability: The incidence can vary significantly depending on the underlying cause of chronic pancreatitis, with alcoholic chronic pancreatitis patients potentially having a higher risk of acute exacerbations.

Underreporting: It's believed that the true incidence may be higher than reported, as some cases might be misclassified as either acute pancreatitis or a non-specific exacerbation of chronic pancreatitis.

Geographical differences: Incidence rates may vary between countries and regions, influenced by factors such as alcohol consumption patterns, genetic predisposition, and healthcare access.

Increasing awareness: As recognition of acute-on-chronic pancreatitis as a distinct entity grows, reported incidence rates may change.

It's important to note that these figures are approximate and may not reflect the most current data. The incidence of acute-on-chronic pancreatitis is an area where more research is needed to establish more precise and up-to-date figures.

Would you like more information on factors that influence the incidence or any related epidemiological aspects of acute-on-chronic pancreatitis?

LIKERT SCORE = ______

Q0503: What is the incidence of acute on chronic pancreatitis?

Chatbot answer: *The incidence of acute pancreatitis ranges from 13 to 45 cases per 100,000 population per year, while chronic pancreatitis ranges from 5 to 12 cases per 100,000 population per year1. Acute pancreatitis can sometimes lead to chronic pancreatitis, with about 20% of people experiencing a recurrence of acute pancreatitis, and 36% of those going on to develop chronic pancreatitis2.*

Is there anything specific you'd like to know about pancreatitis?

LIKERT SCORE = ______

Q0603: What is the incidence of acute on chronic pancreatitis?

Chatbot answer: *Acute on chronic pancreatitis (ACP) is a complex condition that occurs when a person with chronic pancreatitis experiences a sudden worsening of their symptoms. The exact incidence of ACP is difficult to determine, as it can be challenging to differentiate from other acute pancreatitis episodes. However, studies suggest that it is a significant clinical problem, particularly in regions with high rates of chronic pancreatitis.*

Risk factors for ACP include:

- *Alcohol abuse*
- *Gallstones*
- *Hereditary pancreatitis*
- *Hypertriglyceridemia*
- *Autoimmune pancreatitis*

If you or someone you know is experiencing symptoms of pancreatitis, it's important to seek medical attention. Early diagnosis and treatment can help prevent complications and improve outcomes.

Would you like to know more about the symptoms or treatment options for acute on chronic pancreatitis?

LIKERT SCORE = ______

Q0703: What is the incidence of acute on chronic pancreatitis?

Chatbot answer: *The incidence of acute on chronic pancreatitis can vary widely depending on factors like geographic location, population, and specific risk factors. However, it's estimated that about 25-30% of all pancreatitis cases are actually acute episodes occurring in individuals with pre-existing chronic pancreatitis. Please consult specific studies or medical literature for more precise data.*

LIKERT SCORE = _______

QXX04: What are the risk factors for acute on chronic pancreatitis?

Extract from the position statement:

Question 4: What are the risk factors for ACP?

Answer: Alcohol intake is a risk factor for ACP episodes, whereas the contribution of smoking and main pancreatic duct obstruction is not known.

Quality assessment: Very low

Recommendation: Conditional

Agreement: Strong

Commentary: The lack of an agreed definition of ACP makes it difficult to determine whether any data on risk factors are in reference to acute pancreatic inflammation or to an increase in pain or both. Alcohol is known to be associated with inflammatory complications of CP [27] and some authors conclude that alcohol consumption is an independent risk factor for episodes of ACP [37,38]. Alcohol abstinence appears to protect against ACP [39]. There is some evidence to suggest that alcohol preferentially induces ACP rather than AP [40,41]. This evidence suggests that abstinence from alcohol will decrease the number of ACP episodes. Also, smoking may increase the number of "acute exacerbations" (a term used by the Hungarian Pancreatitis Study Group to describe what is assumed to be an ACP, but due to lack of consensus on description this is not clear) [37]. Olesen et al. showed that smoking was associated with development of fibrosis-related complications, but not to ACP [27]. Turner et al. showed that patients often increased their intake of alcohol and coffee prior to an ACP episode [42]. Another possible risk factor for ACP is main pancreatic duct obstruction [43–45] but, again, it is difficult to distinguish between an ACP episode and an aggravation of the underlying chronic pain syndrome without an overt inflammatory response. Well-designed studies, including a clear distinction between episodes of ACP and aggravation of the chronic pain syndrome associated with CP, are needed to evaluate these putative risk factors. While evidence is lacking regarding other risk factors, abstinence and control of all known risk factors for CP in general should be recommended as it may reduce the frequency of ACP episodes and decrease risk of disease progression towards end-stage CP.

Q0104: What are the risk factors for acute on chronic pancreatitis?

Chatbot answer: *Risk factors for acute pancreatitis: alcohol abuse, fatty, spicy, heavy food, long-term use of certain medications, abdominal trauma in the pancreas area, chronic*

gallstone disease, inflammation of other digestive organs, congenital or acquired defects of the pancreas, viral infections, bacterial infections. Risk factors for chronic pancreatitis: alcohol abuse, genetic predisposition, autoimmune diseases, poor nutrition, smoking, obesity, side effects of certain medications, diseases of the gallbladder and bile ducts, viral infections, bacterial infections.

LIKERT SCORE = ______

Q0204: What are the risk factors for acute on chronic pancreatitis?

Chatbot answer: *The development of acute-on-chronic pancreatitis can be influenced by several risk factors. Here are some of the key factors associated with increased risk:*

1. Alcohol Consumption

- *Chronic Alcohol Use: This is one of the most significant risk factors. Patients with a history of chronic alcohol consumption are more likely to experience acute exacerbations of pancreatitis.*

- *Binge Drinking: Episodes of heavy drinking can precipitate acute inflammation.*

2. Smoking

- *Cigarette smoking has been linked to an increased risk of both chronic pancreatitis and episodes of acute-on-chronic pancreatitis. Smoking can exacerbate inflammation and pancreatic damage.*

3. Gallstones

- *Gallstones can cause acute pancreatitis by obstructing the pancreatic duct or the common bile duct, leading to inflammation. In patients with chronic pancreatitis, gallstones can trigger acute episodes.*

4. Pancreatic Duct Obstruction

- *Any condition that causes obstruction of the pancreatic duct (e.g., strictures, tumors) can lead to increased pressure and inflammation, resulting in acute exacerbations.*

5. Dietary Factors

- *High-fat diets and dietary indiscretion can provoke acute attacks in individuals with chronic pancreatitis. Certain foods may exacerbate pancreatic inflammation.*

6. Genetic Factors

- *Hereditary pancreatitis or genetic mutations (e.g., PRSS1, SPINK1, CFTR mutations) can predispose individuals to both chronic and acute-on-chronic pancreatitis.*

7. Metabolic Conditions

- *Conditions such as diabetes mellitus, hyperlipidemia, and hypercalcemia may increase the risk of acute exacerbations.*

8. Infections

• *Infections (e.g., viral infections like mumps or cytomegalovirus) can exacerbate pancreatic inflammation in individuals with chronic pancreatitis.*

9. Recent Surgical Procedures

• *Abdominal surgeries, especially those involving the pancreas or biliary system, may increase the risk of developing acute-on-chronic pancreatitis.*

10. Medication Use

• *Certain medications (e.g., corticosteroids, some diuretics) may be associated with increased risk or exacerbation of pancreatitis.*

Summary

Identifying and managing these risk factors is crucial for preventing acute-on-chronic pancreatitis episodes. Lifestyle modifications, regular monitoring, and appropriate medical management can help mitigate the risks in patients with chronic pancreatitis.

LIKERT SCORE = ______

Q0304: What are the risk factors for acute on chronic pancreatitis?

Chatbot answer: *The risk factors for acute-on-chronic pancreatitis largely overlap with those for chronic pancreatitis, but certain factors can increase the likelihood of an acute exacerbation. Here are the main risk factors:*

1. *Alcohol consumption:*
    - *Continued alcohol use in patients with alcoholic chronic pancreatitis*
    - *Binge drinking episodes*
2. *Smoking:*
    - *Active smoking*
    - *Heavy tobacco use*
3. *Diet:*
    - *High-fat meals*
    - *Poor nutritional status*
4. *Metabolic factors:*
    - *Hypertriglyceridemia*
    - *Hypercalcemia*
5. *Obstruction:*
    - *Pancreatic duct stones*
    - *Strictures in the pancreatic duct*

6. *Genetic factors:*
    - *Mutations in genes like PRSS1, SPINK1, or CFTR*
7. *Autoimmune factors:*
    - *Flares in autoimmune pancreatitis*
8. *Medications:*
    - *Certain drugs known to cause pancreatitis (e.g., azathioprine, 6-mercaptopurine)*
9. *Infections:*
    - *Viral infections (e.g., mumps)*
    - *Bacterial infections leading to sepsis*
10. *Procedural factors:*
    - *Recent endoscopic retrograde cholangiopancreatography (ERCP)*
11. *Comorbidities:*
    - *Poorly controlled diabetes*
    - *Chronic kidney disease*
12. *Environmental factors:*
    - *Exposure to certain toxins or chemicals*
13. *Age and gender:*
    - *Male gender (slightly higher risk)*
    - *Middle-aged to older adults*
14. *Severity of chronic pancreatitis:*
    - *More advanced chronic pancreatitis may predispose to acute exacerbations*

It's important to note that the presence of multiple risk factors can have a cumulative effect, increasing the overall risk of acute-on-chronic pancreatitis. Management often involves addressing modifiable risk factors to reduce the likelihood of acute exacerbations.

Would you like more information on any specific risk factor or how they interact in the development of acute-on-chronic pancreatitis?

LIKERT SCORE = ______

Q0504: What are the risk factors for acute on chronic pancreatitis?

Chatbot answer: *Acute on chronic pancreatitis occurs when an acute episode of inflammation happens in a pancreas already affected by chronic pancreatitis. Here are some key risk factors:*

1. Heavy Alcohol Use: Chronic and excessive alcohol consumption is a major risk factor12.

2. Smoking: Tobacco use increases the risk of both acute and chronic pancreatitis3.

3. Gallstones: These can block the bile duct, leading to acute pancreatitis1.

4. High Triglyceride Levels: Elevated levels of triglycerides in the blood can trigger pancreatitis13.

5. Genetic Factors: A family history of pancreatitis can increase your risk3.

6. Obesity: Being overweight, especially with fat concentrated around the abdomen, is a risk factor3.

7. Diabetes: This condition is associated with an increased risk of pancreatitis3.

8. Certain Medications: Some drugs can induce pancreatitis1.

9. High Calcium Levels: Conditions like hyperparathyroidism that cause high calcium levels can lead to pancreatitis4.

If you have any of these risk factors, it's important to manage them to reduce the risk of pancreatitis. Do you have any specific concerns or symptoms you're experiencing?

LIKERT SCORE = ______

Q0604: What are the risk factors for acute on chronic pancreatitis?

Chatbot answer: *Risk Factors for Acute On Chronic Pancreatitis*

Acute on chronic pancreatitis occurs when a person with chronic pancreatitis experiences a sudden, severe worsening of their condition. Several factors can contribute to this:

Primary Risk Factors:

- *Chronic Pancreatitis: This is the underlying condition that increases the risk of acute exacerbations.*
- *Alcohol Abuse: Continued alcohol consumption, even after the onset of chronic pancreatitis, can trigger acute attacks.*
- *Gallstones: These can block the pancreatic duct and lead to inflammation.*
- *Hypertriglyceridemia: High levels of triglycerides in the blood can also contribute to pancreatitis.*

Secondary Risk Factors:

- *Medications: Certain medications can increase the risk of pancreatitis, such as corticosteroids, diuretics, and some antiviral drugs.*
- *Trauma or Surgery: Injury to the pancreas can trigger acute pancreatitis.*

• *Infections: Viral or bacterial infections can exacerbate existing pancreatic inflammation.*

• *Autoimmune Diseases: Conditions like autoimmune pancreatitis can increase the risk of acute attacks.*

• *Genetic Predisposition: In some cases, genetic factors can play a role in the development of chronic pancreatitis and its complications.*

It's important to note that these are general risk factors, and individual circumstances may vary. If you have concerns about your risk for acute on chronic pancreatitis, it's best to consult with a healthcare professional.

LIKERT SCORE = **______**

Q0704: What are the risk factors for acute on chronic pancreatitis?

Chatbot answer: *1. Heavy alcohol consumption*

2. Gallstones

3. Smoking

4. High fat diet

5. Genetic factors

6. Certain medications

7. Hypertriglyceridemia (high levels of triglycerides in the blood)

8. Hypercalcemia (high levels of calcium in the blood)

9. Autoimmune diseases

10. Trauma to the abdomen

These risk factors can contribute to both the development of chronic pancreatitis and the occurrence of acute episodes in individuals with pre-existing chronic pancreatitis.

LIKERT SCORE = **______**

QXX05: What are the symptoms and signs of acute on chronic pancreatitis?

Extract from the position statement:

Question 5: What are the symptoms and signs of ACP?
Answer: Although pain is the dominant symptom of ACP, non-specific symptoms or signs such as vomiting or jaundice may be seen. Despite being rare, a diagnosis of ACP in the appropriate clinical setting should not be discarded based on the absence of pain.
Quality assessment: Low
Recommendation: Conditional
Agreement: Strong

Commentary: The typical symptoms and signs in patients with ACP overlap with those of AP [46]. The dominant symptom is typically pancreatic type abdominal pain (acute onset, epigastric and often radiating to the mid-back). However, patients with ACP may, in rare cases, present without pain and exhibit atypical or unspecific symptoms and/or signs. Hence, patients may present with vomiting, especially those with groove pancreatitis [47], or with transient jaundice during ACP episodes [48]. This may be due to pancreatic head swelling accompanying the inflammatory process, unlike persistent jaundice of chronic pancreatitis due to fibrosis [49]. There are also case reports of episodes of ACP without abdominal pain, where the patients presented subcutaneous nodules of panniculitis [28,31] or abnormal electrocardiographic findings [29].

Q0105: What are the symptoms and signs of acute on chronic pancreatitis?

Chatbot answer: *Symptoms of acute pancreatitis include intense girdle pain, repeated vomiting, bloating, fever, nausea, and loose stools. Chronic pancreatitis is characterized by long periods of exacerbation, accompanied by pain, bowel disturbances, and weight loss.*

LIKERT SCORE = ______

Q0205: What are the symptoms and signs of acute on chronic pancreatitis?

Chatbot answer: *The symptoms and signs of acute-on-chronic pancreatitis typically mirror those of acute pancreatitis but may also reflect the underlying chronic condition. Here's a detailed overview:*

Symptoms

1. Abdominal Pain:

o Severe Pain: Sudden onset of severe, persistent abdominal pain, often located in the upper abdomen. The pain may radiate to the back.

o Nature of Pain: It may be described as sharp, stabbing, or dull, and often worsens after eating or drinking.

2. Nausea and Vomiting:

o Patients commonly experience nausea and may have episodes of vomiting, which can be persistent.

3. Loss of Appetite:

o Decreased desire to eat due to abdominal pain and nausea.

4. Weight Loss:

o Unintentional weight loss may occur over time due to malabsorption or dietary restrictions.

5. Diarrhea or Steatorrhea:

o Loose, fatty stools (steatorrhea) may occur due to malabsorption of nutrients, particularly fats.

6. Jaundice:

o Yellowing of the skin and eyes may occur if there is bile duct obstruction due to pancreatic swelling or gallstones.

7. Fever:

o Low-grade fever may be present, especially if there is associated infection or inflammation.

Signs

1. Tenderness on Abdominal Examination:

o The abdomen may be tender, particularly in the upper abdominal region.

2. Guarding or Rigidity:

o In severe cases, there may be involuntary guarding (muscle tightness) or rigidity of the abdominal wall.

3. Dehydration Signs:

o Signs of dehydration, such as dry mucous membranes or decreased skin turgor, may be present due to vomiting and inadequate fluid intake.

4. Hypotension and Tachycardia:

o These may occur due to fluid loss and dehydration, particularly in more severe cases.

5. Signs of Shock:

o In severe cases, the patient may exhibit signs of shock, including confusion, lethargy, or decreased urine output.

6. Elevated Heart Rate:

o Tachycardia may be observed as a physiological response to pain or dehydration.

Summary

Acute-on-chronic pancreatitis presents with a combination of acute and chronic symptoms, with severe abdominal pain being a hallmark sign. Early recognition and management of these symptoms are essential to prevent complications and improve patient outcomes. If symptoms arise, it is crucial for individuals to seek medical attention promptly.

LIKERT SCORE = ______

Q0305: What are the symptoms and signs of acute on chronic pancreatitis?

Chatbot answer: *The symptoms and signs of acute-on-chronic pancreatitis are a combination of those seen in acute pancreatitis superimposed on the background of chronic pancreatitis. Here's a comprehensive list:*

Symptoms:

1. Abdominal pain:

o Sudden onset or worsening of pain, typically in the upper abdomen

o Pain may radiate to the back

o Often more severe than the usual chronic pain

2. Nausea and vomiting

3. Loss of appetite

4. Worsening of chronic symptoms:

o Increased frequency or severity of chronic abdominal pain

o Exacerbation of malabsorption symptoms (e.g., diarrhea, steatorrhea)

5. Fatigue and weakness

6. Fever (in some cases)

7. Shortness of breath (in severe cases due to pleural effusions or acute respiratory distress syndrome)

Signs:

1. Physical examination findings:

o Abdominal tenderness, often in the epigastric region

o Abdominal distension

o Decreased bowel sounds

o Jaundice (if biliary obstruction occurs)

2. Vital sign changes:

o Tachycardia (rapid heart rate)

o Hypotension (in severe cases or with significant fluid loss)

o *Fever*

3. *Signs of dehydration:*

o *Dry mucous membranes*

o *Reduced skin turgor*

4. *Complications-related signs:*

o *Grey Turner's sign (bruising in the flanks) or Cullen's sign (periumbilical bruising) in severe cases with retroperitoneal bleeding*

o *Pleural effusion (detectable on chest examination)*

5. *Worsening of pre-existing signs of chronic pancreatitis:*

o *Increased abdominal distension*

o *More prominent signs of malnutrition*

6. *New-onset or worsening diabetes:*

o *Polyuria, polydipsia*

7. *Ascites (in some cases)*

8. *Rarely, palpable abdominal mass (if pseudocyst forms)*

It's important to note that the presentation can vary significantly between individuals. Some patients with chronic pancreatitis may have a diminished pain response due to progressive nerve damage, potentially masking the typical pain of an acute exacerbation. Additionally, the severity of symptoms and signs can range from mild to life-threatening, depending on the extent of the acute inflammation and any resulting complications.

Would you like more information on how these symptoms and signs are evaluated or managed in a clinical setting?

LIKERT SCORE = ______

Q0505: What are the symptoms and signs of acute on chronic pancreatitis?

Chatbot answer: *Acute on chronic pancreatitis combines features of both acute and chronic pancreatitis. Here are some common symptoms and signs:*

Acute Pancreatitis Symptoms:

- *Severe abdominal pain that may radiate to the back*
- *Nausea and vomiting*
- *Fever*
- *Rapid pulse*
- *Swollen and tender abdomen*

Chronic Pancreatitis Symptoms:

- *Persistent upper abdominal pain that may worsen after eating*
- *Weight loss without trying*
- *Oily, smelly stools (steatorrhea)*
- *Diarrhea*
- *Nausea and vomiting*
- *Fatigue*
- *Shortness of breath*

When acute pancreatitis occurs on top of chronic pancreatitis, the symptoms of acute pancreatitis can be more severe and prolonged.

If you or someone you know is experiencing these symptoms, it's important to seek medical attention promptly. Do you have any other questions or need further information?

LIKERT SCORE = ______

Q0605: What are the symptoms and signs of acute on chronic pancreatitis?

Chatbot answer: *Acute on Chronic Pancreatitis: A Double Whammy*

Acute on chronic pancreatitis is a serious condition where a sudden flare-up (acute pancreatitis) occurs in a person who already has chronic pancreatitis. It can be a particularly painful and dangerous situation.

Symptoms and Signs

Both acute and chronic pancreatitis can share similar symptoms, but the combination of the two can be more severe and persistent. Here are some common symptoms:

- *Severe abdominal pain: This is often the most prominent symptom, often radiating to the back.*
- *Nausea and vomiting: These are common, especially after eating.*
- *Fever: A fever is a sign of inflammation and infection.*
- *Weight loss: Difficulty digesting food can lead to unintentional weight loss.*
- *Jaundice: This occurs when the liver is unable to process bilirubin, a pigment produced by the breakdown of red blood cells.*
- *Diarrhea or constipation: Changes in bowel habits are common.*
- *Oily stools: This can be a sign of malabsorption due to pancreatic enzyme deficiency.*

When to Seek Medical Attention

If you experience any of these symptoms, especially if you have a history of chronic pancreatitis, it's important to seek immediate medical attention. Early diagnosis and treatment can help prevent complications.

Please note: This information is intended for general knowledge only and does not constitute medical advice. Always consult with a healthcare professional for personalized guidance.

LIKERT SCORE = ______

Q0705: What are the symptoms and signs of acute on chronic pancreatitis?

Chatbot answer: *1. Severe abdominal pain, often in the upper abdomen radiating to the back*

2. Nausea and vomiting

3. Fever

4. Increased heart rate

5. Decreased appetite

6. Swollen or tender abdomen

7. Tenderness when touching the abdomen

8. Indigestion and malabsorption

9. Fatty stools (steatorrhea)

10. Weight loss

Please note that these symptoms can also be indicative of other conditions. Always consult with a healthcare professional for an accurate diagnosis.

LIKERT SCORE = ______

QXX06: Are serum markers useful for diagnosing acute on chronic pancreatitis?

Extract from the position statement:

Question 6: Are serum markers useful for diagnosing ACP?
Answer: Serum lipase should be preferred as serum marker to aid the diagnose ACP but, in case of unavailability, serum amylase can also be used. Serum levels of both lipase and amylase less than 3 times the upper limit of normal does not exclude ACP. Additional biomarkers might aid in more accurate diagnosis of ACP but require further validation.
Quality assessment: Low
Recommendation: Conditional
Agreement: Strong

Commentary: The diagnosis of AP is based on the Revised Atlanta Classification [7] where two of three criteria are required. These are typical pancreatic pain, an increase in the level of serum amylase or lipase to over three times the upper limit of normal and/or radiological evidence of AP. There is some agreement that smaller increases in serum pancreatic enzymes could be accepted for the diagnosis of ACP [50–55] which is consistent with a decreased acinar cell mass, secondary to the fibroinflammatory process and pancreatic atrophy associated with CP [56]. In a study of ACP patients, serum amylase and lipase levels were increased to at least three times the upper limit of normal in only 20% and 60% of episodes and were within normal range in 36% and 24%, respectively [5]. Despite these findings, the panel consider that there is insufficient evidence to support a lower diagnostic threshold from that used in AP (Fig. 2). While both amylase and lipase levels can be within normal range in ACP, it appears that lipase is a more sensitive diagnostic marker of ACP [53]. This is supported by the finding that CP tissue shows a greater decline in amylase activity compared with lipase activity (91% vs 26%) [51]. This is also supported by studies suggesting that a lipase/amylase ratio > 2–3 is indicative of ACP [57,58] and points to lipase being more accurate for diagnosing ACP.

Other biomarkers might contribute to the diagnosis of ACP, but they all require further validation. Urine trypsinogen was found to be useful for identifying pancreatitis in patients with pancreatic insufficiency in the presence of normal amylase or lipase levels [59]. The value of this biomarker in ACP is not well established. Other serum markers include carbohydrate antigen 19-9 (CA 19-9) which can be elevated in "acute exacerbations" of CP [60], but can also be elevated in cancer and cholangitis. Because CA 19-9 is not specific for ACP and 10% of the population cannot express this biomarker, it is unlikely to be useful as a diagnostic marker for ACP. It has been found that the acute phase protein ceruloplasmin can be significantly increased in ACP [61], probably reflecting the acute inflammatory process in the pancreas. It has also been noted that there is a close correlation between marked eosinophilia and ACP, but the diagnostic value has yet to be determined [62]. Other inflammatory markers such as C reactive protein and pro-inflammatory cytokines have not been shown to increase the accuracy of diagnosing ACP.

Q0106: Are serum markers useful for diagnosing acute on chronic pancreatitis?

Chatbot answer: *Yes, serum markers are useful in the diagnosis of acute and chronic pancreatitis. They include cytokines (e.g., IL-6, IL-8), pancreatic secretions (phospholipase A2, serine protease inhibitor type 1), and other markers (tumor necrosis factor, soluble TNF-a receptors). These markers help assess the severity, predict outcome, and monitor the effectiveness of treatment for pancreatitis.*

LIKERT SCORE = ______

Q0206: Are serum markers useful for diagnosing acute on chronic pancreatitis?

Chatbot answer: *Serum markers can be useful in diagnosing acute-on-chronic pancreatitis, but they must be interpreted in the context of the patient's history, clinical presentation, and imaging studies. Here are some key serum markers and their relevance:*

1. Serum Amylase and Lipase

- *Amylase: Serum amylase levels are often elevated in cases of acute pancreatitis. However, in chronic pancreatitis, the levels may be normal or only mildly elevated.*
- *Lipase: Serum lipase is more specific for pancreatic injury and typically remains elevated for a longer duration than amylase. Elevated lipase levels are a strong indicator of acute pancreatitis, including acute-on-chronic episodes.*

2. C-Reactive Protein (CRP)

- *CRP is an acute-phase reactant that can indicate inflammation. Elevated CRP levels may correlate with the severity of pancreatitis and can help assess the inflammatory response during an acute exacerbation.*

3. White Blood Cell Count (WBC)

- *An elevated white blood cell count can indicate inflammation and infection. In acute-on-chronic pancreatitis, leukocytosis may be present, reflecting an inflammatory response.*

4. Blood Glucose Levels

- *Hyperglycemia can occur due to pancreatic damage and is more common in patients with chronic pancreatitis. This can indicate a loss of insulin-producing capability.*

5. Bilirubin

- *Elevated serum bilirubin levels may indicate bile duct obstruction due to inflammation or pancreatic swelling, especially in cases of acute-on-chronic pancreatitis associated with gallstones.*

6. Liver Function Tests

- *Abnormal liver function tests (elevated transaminases, alkaline phosphatase) may indicate biliary involvement or complications related to the pancreas.*

Limitations

- *Sensitivity and Specificity: While elevated amylase and lipase are indicative of pancreatitis, they are not specific to acute-on-chronic pancreatitis, as these markers can also be elevated in other conditions (e.g., renal failure, bowel obstruction).*
- *Chronic Pancreatitis: In patients with chronic pancreatitis, baseline levels of amylase and lipase may be normal or not significantly elevated during acute episodes, making diagnosis challenging.*

Conclusion

Serum markers such as lipase, CRP, WBC count, bilirubin, and liver function tests can support the diagnosis of acute-on-chronic pancreatitis. However, they should be used in conjunction with clinical evaluation and imaging studies to establish a definitive diagnosis.

LIKERT SCORE = ______

Q0306: Are serum markers useful for diagnosing acute on chronic pancreatitis?

Chatbot answer: *Serum markers can be useful in diagnosing acute-on-chronic pancreatitis, but they have some limitations. Here's an overview of the key serum markers and their utility:*

1. Pancreatic enzymes:

- Amylase: Often elevated, but may be less reliable in chronic pancreatitis due to decreased pancreatic function.

- Lipase: Generally more specific and sensitive than amylase. An elevation of 3 times the upper limit of normal is typically considered diagnostic for acute pancreatitis.

Utility: These are the primary serum markers used. However, in chronic pancreatitis, enzyme elevations may be less pronounced due to reduced pancreatic mass.

2. Inflammatory markers:

- C-reactive protein (CRP): Elevated in acute inflammation.

- Procalcitonin: Can help distinguish between infected and sterile pancreatic necrosis.

Utility: These can indicate the presence and severity of acute inflammation, but are not specific to pancreatitis.

3. Liver function tests:

- Alanine aminotransferase (ALT), Aspartate aminotransferase (AST), Alkaline phosphatase (ALP), and Bilirubin.

Utility: Can help identify biliary causes or complications.

4. Triglycerides:

- Useful in identifying hypertriglyceridemia as a potential cause.

5. Calcium levels:

- To rule out hypercalcemia as a cause.

6. Glucose levels:

- May be elevated due to pancreatic dysfunction.

Limitations and considerations:

1. Sensitivity issues: In chronic pancreatitis, serum enzyme levels may not rise as high as in acute pancreatitis due to reduced functional pancreatic tissue.

2. Specificity: Many of these markers can be elevated in other conditions, requiring clinical correlation.

3. Timing: Enzyme levels may normalize quickly, so timing of the test is crucial.

4. Variability: The degree of elevation doesn't always correlate with the severity of the disease.

5. False negatives: Some patients with acute-on-chronic pancreatitis may not show significant elevations in serum markers.

6. Need for trending: Serial measurements may be more informative than a single value.

In conclusion, while serum markers are useful in diagnosing acute-on-chronic pancreatitis, they should be interpreted in the context of clinical presentation, imaging findings, and the patient's history. They are most useful when combined with other diagnostic modalities rather than used in isolation.

Would you like more information on how these serum markers are used in clinical decision-making or their comparative effectiveness?

LIKERT SCORE = ______

Q0506: Are serum markers useful for diagnosing acute on chronic pancreatitis?

Chatbot answer: *Serum markers, such as amylase and lipase, are commonly used in diagnosing acute pancreatitis. These markers typically rise within hours of pancreatic injury and can remain elevated for several days. However, their utility in diagnosing chronic pancreatitis, or acute on chronic pancreatitis, is more limited.*

In chronic pancreatitis, serum levels of amylase and lipase may be only mildly elevated or even normal, especially as the pancreas becomes more fibrotic over time. This makes them less reliable for diagnosing chronic conditions. Additionally, elevated levels of these enzymes can be seen in various other conditions, reducing their specificity for chronic pancreatitis.

For a more accurate diagnosis of chronic pancreatitis, imaging studies (such as CT scans, MRI, or endoscopic ultrasound) and functional tests are often required.

If you have any more questions or need further clarification, feel free to ask!

LIKERT SCORE = ______

Q0606: Are serum markers useful for diagnosing acute on chronic pancreatitis?

Chatbot answer: *Serum Markers for Acute-on-Chronic Pancreatitis: A Valuable Diagnostic Tool*

Yes, serum markers can be useful in diagnosing acute-on-chronic pancreatitis. While not definitive on their own, they can provide valuable information when combined with clinical presentation and imaging studies.

Common Serum Markers:

- *Lipase and amylase: These enzymes are typically elevated in both acute and chronic pancreatitis. However, their levels may be less elevated or return to normal more quickly in acute-on-chronic pancreatitis compared to acute pancreatitis.*
- *C-reactive protein (CRP): A marker of inflammation, CRP is often elevated in acute-on-chronic pancreatitis.*
- *Interleukin-6 (IL-6): Another inflammatory marker, IL-6 can also be elevated in this condition.*
- *Other markers: Some studies have explored the use of additional markers, such as pancreatic trypsinogen activation peptide (PTAP) and regenerating islet-derived protein 3-alpha (REG3α), as potential diagnostic aids for acute-on-chronic pancreatitis.*

Limitations:

- *Overlapping elevations: Serum markers can sometimes be elevated in other conditions, such as acute pancreatitis, biliary tract disease, or gastrointestinal perforation.*
- *Lack of specificity: No single serum marker is completely specific for acute-on-chronic pancreatitis.*
- *Individual variability: The levels of these markers can vary significantly between individuals.*

It's important to note that a diagnosis of acute-on-chronic pancreatitis should be based on a combination of clinical, laboratory, and imaging findings. Serum markers can play a valuable role in this diagnostic process, but they should not be relied upon in isolation.

Would you like to know more about the symptoms or treatment of acute-on-chronic pancreatitis?

LIKERT SCORE = ______

Q0706: Are serum markers useful for diagnosing acute on chronic pancreatitis?

Chatbot answer: *Yes, serum markers can be useful in diagnosing acute on chronic pancreatitis. The two most commonly used are:*

1. *Amylase: This is an enzyme that helps digest carbohydrates. Its levels usually increase within 12 to 48 hours of an acute pancreatitis attack and then return to normal within a few days.*
2. *Lipase: This is another enzyme that aids in digestion, particularly of fats. Its levels rise more slowly than amylase but remain elevated for a longer period. Lipase is more specific to the pancreas than amylase, making it a more reliable marker.*

However, these markers should be used in conjunction with other diagnostic tools, such as imaging tests and clinical symptoms, for a definitive diagnosis.

LIKERT SCORE = ______

<u>QXX07: Is cross-sectional imaging useful to diagnose acute on chronic pancreatitis in a patient with chronic pancreatitis and worsening of pain?</u>

Extract from the position statement:

Question 7: Is cross-sectional imaging useful to diagnose ACP in a patient with CP and worsening of pain?

Answer: Cross-sectional imaging should be used to support the diagnosis of ACP and rule out complications or other conditions in CP patients.
Quality assessment: Low
Recommendation: Conditional
Agreement: Strong

Commentary: Worsening pain in a patient with CP can be due to ACP, but might also be due to a complication of CP (e.g., development of a pseudocyst, ductal obstruction from a stone or stricture, inflammatory mass or malignancy), other diseases causing abdominal pain unrelated to CP or due to an increase in pain associated with a chronic pain syndrome. Cross-sectional imaging is indicated when there is a strong clinical suspicion of ACP and pancreatic enzymes are <3 times upper limit of normal or when complications of CP are suspected. This approach differs from the recommendation in AP [7], where cross-sectional imaging is not usually recommended in the early phase of uncomplicated AP. In patients with CP and suspicion of ACP, the most appropriate imaging modality is contrast enhanced computed tomography (CT) scan [63]. Magnetic resonance imaging can also be used to diagnose/rule out ACP and address ductal/cystic and potential necrotic inflammatory changes in more details. Ultrasonography is used in some centres to diagnose ACP, but this is highly operator-dependent and often of limited valued due to bowel gas shadows obscuring some or all of the pancreas [64]. Endoscopic ultrasonography can overcome this limitation but is invasive and can be unpleasant for patients, especially in the acute phase, and requires considerable expertise which hinders a wider application. To improve the accuracy of diagnosing ACP it is important to compare any images with previous studies to establish that the findings observed represent acute inflammatory changes in a patient with pre-existing CP morphology. If cross-sectional imaging does not suggest ACP, signs of complications of CP or other abdominal pathology, a worsening of the chronic pain syndrome (without overt inflammation) should be assumed and the term “painful exacerbation of CP” should be used rather than ACP (Fig. 3).

Q0107: Is cross-sectional imaging useful to diagnose acute on chronic pancreatitis in a patient with chronic pancreatitis and worsening of pain?

Chatbot answer: *Yes, cross-sectional imaging is useful for diagnosing acute or chronic pancreatitis in a patient with chronic pancreatitis and worsening pain. It allows evaluation of the pancreas and possible changes associated with pancreatitis.*

LIKERT SCORE = ______

Q0207: Is cross-sectional imaging useful to diagnose acute on chronic pancreatitis in a patient with chronic pancreatitis and worsening of pain?

Chatbot answer: *Yes, cross-sectional imaging is very useful for diagnosing acute-on-chronic pancreatitis in patients with chronic pancreatitis who are experiencing a worsening of pain. Here's how imaging plays a critical role:*

1. Identification of Complications

Cross-sectional imaging, particularly computed tomography (CT) and magnetic resonance imaging (MRI), can help identify complications associated with acute-on-chronic pancreatitis, including:

- *Pancreatic necrosis: Areas of non-viable pancreatic tissue.*
- *Pancreatic fluid collections: Such as pseudocysts or abscesses.*
- *Biliary obstruction: Caused by inflammation or swelling of the pancreas compressing the bile duct.*
- *Vascular complications: Such as thrombosis of the splenic vein.*

2. Assessment of Pancreatic Structure

- *Imaging can reveal changes in pancreatic morphology due to chronic pancreatitis, such as:*
  - o *Dilation or strictures of the pancreatic duct.*
  - o *Calcifications within the pancreas.*
  - o *Altered pancreatic size or shape.*

3. Differentiation from Other Conditions

- *Cross-sectional imaging helps differentiate acute-on-chronic pancreatitis from other conditions that may present with similar symptoms, such as:*
  - o *Cholecystitis or biliary colic.*
  - o *Gastric or duodenal ulcers.*
  - o *Pancreatic tumors or neoplasms.*

4. Detection of Acute Inflammation

- *Imaging can show inflammatory changes around the pancreas, indicating an acute inflammatory episode on top of chronic pancreatitis.*
- *Findings such as edema in the peritoneal cavity or around the pancreas can also support the diagnosis of an acute exacerbation.*

5. Non-Invasive Approach

- *Cross-sectional imaging is non-invasive and can be performed relatively quickly, making it a practical choice for assessing patients with worsening abdominal pain.*

Recommended Imaging Modalities

1. *Computed Tomography (CT):*

o A CT scan is often the first-line imaging modality for acute pancreatitis and can provide detailed information about the pancreas and surrounding structures.

o CT can visualize complications like necrosis, fluid collections, and inflammation.

2. Magnetic Resonance Imaging (MRI):

o MRI may be particularly useful when evaluating complications or when CT is contraindicated.

o MRCP (Magnetic Resonance Cholangiopancreatography) can assess the pancreatic duct and biliary system without contrast.

3. Ultrasound:

o While less sensitive for diagnosing acute-on-chronic pancreatitis, abdominal ultrasound can help evaluate for gallstones or biliary obstruction.

Conclusion

Cross-sectional imaging is a valuable tool in diagnosing acute-on-chronic pancreatitis in patients with a history of chronic pancreatitis and worsening pain. It provides critical information regarding complications, pancreatic structure, and differential diagnoses, guiding appropriate management and intervention.

LIKERT SCORE = ______

Q0307: Is cross-sectional imaging useful to diagnose acute on chronic pancreatitis in a patient with chronic pancreatitis and worsening of pain?

Chatbot answer: *Cross-sectional imaging is indeed very useful in diagnosing acute-on-chronic pancreatitis in a patient with known chronic pancreatitis who is experiencing worsening pain. It plays a crucial role in both confirming the diagnosis and assessing the severity and complications of the acute exacerbation. Here's a detailed explanation of its utility:*

1. Diagnostic value:

- Can differentiate acute exacerbation from other causes of pain in chronic pancreatitis

- Helps visualize acute inflammatory changes superimposed on chronic pancreatic alterations

2. Preferred modalities:

- Contrast-enhanced CT (CECT): Usually the first-line imaging method

- MRI/MRCP: Provides excellent soft tissue contrast and can better visualize ductal anatomy

3. Key findings indicative of acute-on-chronic pancreatitis:

- Pancreatic enlargement or edema

- Peripancreatic fat stranding

- Fluid collections or pseudocysts

- Areas of necrosis (if present)

- These acute changes are seen alongside chronic features like calcifications, atrophy, or ductal dilatation

4. Assessment of complications:

- Vascular complications (e.g., pseudoaneurysms, venous thrombosis)

- Biliary or duodenal obstruction

- Extent of pancreatic necrosis

- Presence and extent of fluid collections or pseudocysts

5. Severity grading:

- Helps in assessing the severity of the acute exacerbation

- Guides management decisions and prognosis

6. Exclusion of other causes:

- Can help rule out other causes of abdominal pain (e.g., bowel obstruction, perforation)

7. Baseline for follow-up:

- Provides a baseline for comparison in subsequent imaging to assess disease progression or treatment response

8. Guidance for interventions:

- If needed, can guide drainage procedures or other interventions

Limitations to consider:

- Radiation exposure with CT (less of an issue with MRI)

- Cost and availability, especially for MRI

- Potential for contrast-related complications

- May not detect very early changes in the acute phase (first 12-24 hours)

In summary, cross-sectional imaging is highly valuable in diagnosing acute-on-chronic pancreatitis, especially in patients with known chronic pancreatitis experiencing worsening pain. It not only confirms the diagnosis but also provides crucial information about severity, complications, and potential need for intervention. The choice between CT and MRI often depends on local availability, patient factors, and the specific clinical question at hand.

Would you like more information on specific imaging protocols or how imaging findings correlate with clinical management decisions in acute-on-chronic pancreatitis?

LIKERT SCORE = ______

Q0507: Is cross-sectional imaging useful to diagnose acute on chronic pancreatitis in a patient with chronic pancreatitis and worsening of pain?

Chatbot answer: *Yes, cross-sectional imaging can be very useful in diagnosing acute on chronic pancreatitis, especially in patients with chronic pancreatitis who are experiencing worsening pain. Techniques like CT (computed tomography) and MRI (magnetic resonance imaging), including MR cholangiopancreatography (MRCP), are commonly used for this purpose12.*

These imaging methods help in identifying structural changes in the pancreas, such as inflammation, fibrosis, and complications like pseudocysts or ductal obstructions23. They can also help differentiate between chronic pancreatitis and acute exacerbations, which is crucial for appropriate management3.

If you have any more questions or need further details, feel free to ask!

LIKERT SCORE = ______

Q0607: Is cross-sectional imaging useful to diagnose acute on chronic pancreatitis in a patient with chronic pancreatitis and worsening of pain?

Chatbot answer: *Yes, cross-sectional imaging is valuable in diagnosing acute-on-chronic pancreatitis in a patient with chronic pancreatitis and worsening pain.*

Here's why:

- *Identifying acute exacerbations: Imaging can reveal signs of acute inflammation, such as pancreatic enlargement, fluid collections, or changes in tissue texture, even in the context of existing chronic damage.*
- *Assessing severity: The extent of the acute inflammation can be evaluated, helping to determine the severity of the exacerbation and guide treatment decisions.*
- *Detecting complications: Imaging can identify potential complications, such as pseudocysts, abscesses, or pancreatic necrosis, which may require specific interventions.*

Common imaging modalities used include:

- *Computed tomography (CT): Offers rapid evaluation of the pancreas and surrounding structures, including the detection of fluid collections and calcifications.*
- *Magnetic resonance imaging (MRI): Provides excellent soft tissue contrast, allowing for detailed assessment of pancreatic structure and function, especially in cases of suspected ductal abnormalities.*

It's important to consult with a healthcare professional for a comprehensive evaluation, as the choice of imaging modality may depend on factors such as the patient's clinical presentation, medical history, and availability of resources.

LIKERT SCORE = ______

Q0707: Is cross-sectional imaging useful to diagnose acute on chronic pancreatitis in a patient with chronic pancreatitis and worsening of pain?

Chatbot answer: *Yes, cross-sectional imaging can be very useful in diagnosing acute on chronic pancreatitis, especially in a patient with chronic pancreatitis who is experiencing worsening of pain. Imaging tests like computed tomography (CT) or magnetic resonance imaging (MRI) can provide valuable information about the pancreas and the surrounding tissues. They can help detect signs of inflammation, such as swelling or fluid collection, and can also reveal any complications, such as necrosis or abscesses. Therefore, these tests are often used to confirm the diagnosis and guide treatment decisions.*

LIKERT SCORE = ______

QXX08: Is cross-sectional imaging more accurate than serum levels of pancreatic enzymes for diagnosing acute on chronic pancreatitis?

Extract from the position statement:

Question 8: Is cross-sectional imaging more accurate than serum levels of pancreatic enzymes for diagnosing ACP?

Answer: Cross-sectional imaging is more accurate than serum levels of pancreatic enzymes for the diagnosis of ACP.
Quality assessment: Very Low
Recommendation: Conditional
Agreement: Strong

Commentary: It is well recognized that pancreatic enzymes can be elevated for many reasons other than ACP [65] and ACP may not result in the same degree of elevation of pancreatic enzymes as in AP (see question 6). Cross-sectional imaging in patients with ACP is able to detect acute inflammatory pancreatic changes, including increased pancreatic and peri-pancreatic oedema, inflammation in surrounding tissue ('dirty fat'), inflammatory masses, acute fluid collections, acute splanchnic thrombosis (including splenic, portal and/or superior mesenteric veins) and can be used to assess the severity of ACP [63] (Fig. 4). Importantly, when radiological criteria of CP are present, cross-sectional imaging enable the distinction between ACP from AP.

Notwithstanding the need for imaging to diagnose ACP in the absence of elevated pancreatic enzymes, any request for a contrast-enhanced CT scan should take into consideration the clinical picture of the patient and recent observations, to balance the correct diagnosis against the risk of excessive radiation exposure. Since some patients experience frequent exacerbations of symptoms for which they are referred to the hospital, cross-sectional imaging should only be pursued when a definitive diagnosis and severity assessment of ACP will alter the treatment approach. Hence, we suggest avoiding repetitive CT scans, in recently admitted patients, when symptoms are similar to recent admissions and appropriate imaging has confirmed an ACP and excluded other complications.

Q0108: Is cross-sectional imaging more accurate than serum levels of pancreatic enzymes for diagnosing acute on chronic pancreatitis?

Chatbot answer: *Cross-sectional imaging may be a more accurate diagnostic method than serum pancreatic enzyme levels, particularly for detecting structural changes in the pancreas. However, both methods have their advantages and disadvantages, and the choice between them depends on the specific clinical situation and the physician's preference.*

LIKERT SCORE = ______

Q0208: Is cross-sectional imaging more accurate than serum levels of pancreatic enzymes for diagnosing acute on chronic pancreatitis?

Chatbot answer: *Yes, cross-sectional imaging is generally considered more accurate than serum levels of pancreatic enzymes for diagnosing acute-on-chronic pancreatitis. Here's a detailed comparison:*

1. Cross-Sectional Imaging

• *Detects Structural Changes: Imaging modalities like CT scans or MRI can visualize structural changes in the pancreas and surrounding tissues, helping to identify complications such as:*

o *Pancreatic necrosis*

o *Fluid collections (pseudocysts or abscesses)*

o *Biliary obstruction*

• *Identifies Inflammation: Imaging can show signs of inflammation around the pancreas, such as edema or inflammatory fluid collections.*

• *Differentiates Conditions: Cross-sectional imaging helps differentiate acute-on-chronic pancreatitis from other abdominal conditions (e.g., cholecystitis, perforated ulcers) that may present similarly.*

• *Non-Invasive: Imaging is non-invasive and provides immediate information about the patient's condition.*

2. Serum Levels of Pancreatic Enzymes

• *Amylase and Lipase:*

o *Specificity and Sensitivity: Elevated serum levels of amylase and lipase are indicative of acute pancreatitis, but they are not specific to acute-on-chronic pancreatitis. In chronic pancreatitis, baseline levels may be normal or only mildly elevated during acute exacerbations.*

o *Timing: Serum amylase levels typically rise within hours of the onset of symptoms but may normalize quickly. Lipase levels remain elevated for a longer duration but may still be insufficient for diagnosis, especially in chronic cases.*

• *Limitations: Elevated enzyme levels can occur in other conditions (e.g., renal failure, bowel obstruction), which can lead to false positives and make it challenging to ascertain the exact nature of the pancreatic inflammation.*

3. Overall Accuracy

• *Imaging vs. Enzymes:*

o *Cross-sectional imaging has the advantage of providing a direct assessment of the pancreas and surrounding structures, allowing for a more comprehensive evaluation of both the extent of disease and any complications.*

o *Serum enzyme levels, while helpful, primarily indicate the presence of pancreatic injury rather than provide specific information regarding the acute-on-chronic nature of the disease.*

Conclusion

In summary, while serum levels of pancreatic enzymes can provide valuable information in diagnosing acute pancreatitis, cross-sectional imaging is more accurate and reliable for diagnosing acute-on-chronic pancreatitis. It not only helps confirm the diagnosis but also assesses the extent of the disease and identifies any complications that may require intervention. For a definitive diagnosis, a combination of both imaging and laboratory tests is often utilized in clinical practice.

LIKERT SCORE = ______

Q0308: Is cross-sectional imaging more accurate than serum levels of pancreatic enzymes for diagnosing acute on chronic pancreatitis?

Chatbot answer: *Cross-sectional imaging is generally considered more accurate than serum levels of pancreatic enzymes for diagnosing acute-on-chronic pancreatitis. Here's a comparative analysis:*

Accuracy of cross-sectional imaging:

1. Sensitivity: High (85-95% for CT, potentially higher for MRI)

2. Specificity: High (90-100%)

3. Provides direct visualization of pancreatic and peripancreatic changes

Accuracy of serum pancreatic enzymes:

1. Sensitivity: Variable (70-100% for lipase, lower for amylase)

2. Specificity: Moderate to high (about 80-100%, higher for lipase)

3. Indirect indicator of pancreatic inflammation

Factors favoring cross-sectional imaging:

1. Visualization of pancreatic morphology:

- Imaging can directly show both chronic changes and acute inflammatory signs

- Allows differentiation between acute exacerbation and chronic pain without acute changes

2. Enzyme level variability in chronic pancreatitis:

- Patients with chronic pancreatitis may have reduced enzyme production

- Can lead to false negatives or less pronounced elevations in acute exacerbations

3. Assessment of complications:

- Imaging can detect and characterize complications that enzyme levels cannot

4. Differential diagnosis:

- Imaging can rule out other causes of abdominal pain

5. Severity assessment:

- Provides information on the extent of inflammation and necrosis

6. Guidance for management:

- Offers valuable information for treatment planning and potential interventions

Limitations of relying solely on enzyme levels:

1. Lack of specificity:

- Enzymes can be elevated in non-pancreatic conditions

2. Timing issues:

- Enzyme levels may normalize quickly, potentially missing the diagnostic window

3. Limited information:

- Don't provide details about the extent of inflammation or complications

4. Chronic pancreatitis effect:

- Enzyme elevations may be less pronounced due to reduced functional pancreatic tissue

However, it's important to note:

1. Complementary use:

- In practice, both imaging and enzyme levels are often used together for a comprehensive evaluation

2. Initial screening:

- Enzyme levels are often checked first due to ease and speed of testing

3. Resource considerations:

- Imaging is more expensive and less immediately available than blood tests

In conclusion, while both have their roles, cross-sectional imaging is generally more accurate and informative for diagnosing acute-on-chronic pancreatitis, especially in patients with known chronic pancreatitis. The visual information provided by imaging, combined with its ability to assess complications and severity, makes it superior to enzyme levels alone. However, the optimal approach often involves using both methods in conjunction for a comprehensive diagnostic evaluation.

Would you like more information on specific imaging findings or how the combination of imaging and laboratory tests is used in clinical decision-making?

LIKERT SCORE = ______

Q0508: Is cross-sectional imaging more accurate than serum levels of pancreatic enzymes for diagnosing acute on chronic pancreatitis?

Chatbot answer: *Yes, cross-sectional imaging is generally considered more accurate than serum levels of pancreatic enzymes for diagnosing acute on chronic pancreatitis.*

Cross-sectional imaging techniques such as contrast-enhanced computed tomography (CT), magnetic resonance imaging (MRI), and endoscopic ultrasonography provide detailed visualizations of the pancreas, allowing for the identification of structural changes, inflammation, fibrosis, and complications12. These imaging methods are particularly useful in detecting chronic pancreatitis, where structural changes are more pronounced.

On the other hand, serum levels of pancreatic enzymes like amylase and lipase can be elevated in acute pancreatitis but are less reliable for chronic conditions. These enzyme levels can also be influenced by other factors and conditions, leading to potential false positives or negatives34.

In summary, while serum enzyme levels are useful for initial assessment, cross-sectional imaging offers a more comprehensive and accurate diagnosis, especially for chronic pancreatitis and its complications.

Is there anything specific you would like to know more about regarding this topic?

LIKERT SCORE = ______

Q0608: Is cross-sectional imaging more accurate than serum levels of pancreatic enzymes for diagnosing acute on chronic pancreatitis?

Chatbot answer: *Cross-sectional imaging is generally considered more accurate than serum levels of pancreatic enzymes for diagnosing acute on chronic pancreatitis (AOP).*

Here's why:

- *Specificity: Cross-sectional imaging, such as CT or MRI, can directly visualize changes in the pancreas, including inflammation, scarring, and calcifications. This provides a more specific diagnosis compared to elevated enzyme levels, which can also be elevated in other conditions.*
- *Severity Assessment: Imaging can help assess the severity of pancreatitis by revealing the extent of pancreatic damage, presence of complications (e.g., pseudocyst formation), and potential underlying causes (e.g., gallstones, alcohol abuse).*
- *Long-term Monitoring: Cross-sectional imaging is valuable for monitoring the progression of chronic pancreatitis, detecting early signs of complications, and evaluating the effectiveness of treatment.*

While elevated serum levels of pancreatic enzymes (lipase and amylase) are often used as initial markers of pancreatitis, they can be less reliable in differentiating between acute and chronic pancreatitis, especially in later stages.

Therefore, cross-sectional imaging is typically recommended as the primary diagnostic tool for AOP. However, it's important to note that the choice of imaging modality and the specific tests performed may vary depending on the clinical context and the availability of resources.

Would you like to know more about the specific types of cross-sectional imaging used for pancreatitis diagnosis?

LIKERT SCORE = ______

Q0708: Is cross-sectional imaging more accurate than serum levels of pancreatic enzymes for diagnosing acute on chronic pancreatitis?

Chatbot answer: *While serum levels of pancreatic enzymes like amylase and lipase can provide useful information in diagnosing acute pancreatitis, they are not as accurate as cross-sectional imaging for diagnosing acute on chronic pancreatitis. This is because serum enzyme levels can be normal or only mildly elevated in chronic pancreatitis, and they may not reliably indicate an acute exacerbation.*

On the other hand, cross-sectional imaging can directly visualize the pancreas and detect signs of inflammation or complications, providing a more accurate and definitive diagnosis. However, it's important to note that imaging tests should be used in conjunction with clinical symptoms and other diagnostic tools for a comprehensive evaluation.

Extract from the position statement:

LIKERT SCORE = ______

QXX09: Is the severity of acute on chronic pancreatitis comparable to acute pancreatitis in patients without chronic pancreatitis?

Extract from the position statement:

Question 9: Is the severity of ACP comparable to AP in patients without CP?

Answer: ACP tends to be less severe compared with AP.
Quality assessment: Very Low
Recommendation: Conditional
Agreement: Strong

Commentary: According to the Revised Atlanta classification [7], severity is based on presence of organ failure (transient or persistent), local complications (peripancreatic acute fluid collections, acute necrotic collections, pseudocyst and walled off necrosis) or systemic complications (exacerbations of underlying comorbidities). These factors have not been systematically studied in patients with ACP and, as a result, there are no specific guidelines or severity classification that can be applied to patients with ACP. By default, there is a tendency to use the categories of severity for AP even though these were not designed or validated for this purpose.

There is a common understanding that patients with ACP have a more favourable outcome compared to patients with AP [66–71]. It is assumed that pancreatic fibrosis has a role in limiting acinar cell necrosis [72,73]. Factors associated with increased severity in ACP are age, comorbidity and weight loss [33]. According to Akshintala et al., patients with ACP have lower rates of multisystem organ failure, need for intensive care unit, and mortality [33]. However, in a study from Weitz et al. comparing ACP with AP, renal failure and the need for intensive care unit were more frequent in patients with ACP, even though there was a lower rate of severe episodes in that cohort [68]. There are a number of pancreas related complications that are common in ACP. For example, in a study from Kim and Kim [34], the incidence of pancreatic pseudocysts in ACP was 41.8%, compared with 14.6% in AP. The higher incidence of pseudocysts in ACP is probably due to the fact that they often are chronic pseudocysts that usually persists for very long periods in CP. There is also a consensus among experts, that necrotic parenchyma is rarely seen on a background of well-established CP and, since the imaging changes seen in ACP are typically mild with only minimal necrosis, the use of the Revised Atlanta Classification [7] is often not appropriate. Furthermore, ACP is not expected to be seen in end-stage CP where the inflammation disappears and only the fibrotic process remains. Further research is required to evaluate the rate of organ failure, systemic and local complications in ACP.

Q0109: Is the severity of acute on chronic pancreatitis comparable to acute pancreatitis in patients without chronic pancreatitis?

Chatbot answer: *Yes, it is comparable. Acute pancreatitis can occur both in patients with chronic pancreatitis and in those who have not previously suffered from this disease.*

LIKERT SCORE = ______

Q0209: Is the severity of acute on chronic pancreatitis comparable to acute pancreatitis in patients without chronic pancreatitis?

Chatbot answer: *The severity of acute-on-chronic pancreatitis is generally not directly comparable to that of acute pancreatitis in patients without chronic pancreatitis, as there are*

important differences in pathophysiology, clinical presentation, and outcomes. Here are some key points to consider:

1. Pathophysiology

- *Acute Pancreatitis: In individuals without chronic pancreatitis, acute pancreatitis typically arises due to identifiable triggers such as gallstones, alcohol consumption, or hyperlipidemia. The inflammation is usually isolated to the pancreas and can lead to complications, but the pancreas is structurally normal prior to the acute event.*

- *Acute-on-Chronic Pancreatitis: In patients with chronic pancreatitis, the pancreas has already sustained damage and changes (e.g., fibrosis, calcifications). Acute exacerbations occur against a backdrop of pre-existing pancreatic dysfunction and may lead to more severe inflammatory responses.*

2. Severity Assessment

- *Severity Grading: Both acute pancreatitis and acute-on-chronic pancreatitis can be graded based on severity (mild, moderate, severe) using scoring systems like the Revised Atlanta Classification. However, the baseline condition of the pancreas in chronic cases often means that the same level of acute inflammatory response can lead to worse outcomes.*

- *Complications: Acute-on-chronic pancreatitis may be associated with a higher risk of severe complications, such as:*

o *Pancreatic necrosis*

o *Infection*

o *Organ failure (due to more systemic involvement)*

o *Increased risk of pseudocyst formation*

3. Clinical Presentation

- *Symptoms: Patients with acute-on-chronic pancreatitis may experience more severe and prolonged symptoms due to the underlying chronic condition and the effects of acute inflammation.*

- *Management Challenges: Management of acute-on-chronic pancreatitis can be more complex because patients may already have functional impairments and complications related to chronic pancreatitis, necessitating more intensive care and interventions.*

4. Outcomes

- *Prognosis: Studies indicate that acute-on-chronic pancreatitis often has a worse prognosis compared to acute pancreatitis alone. Patients with chronic pancreatitis may have a higher risk of developing complications, prolonged hospital stays, and increased mortality rates.*

Conclusion

In summary, while both acute-on-chronic pancreatitis and acute pancreatitis can be severe, the severity and complications associated with acute-on-chronic pancreatitis are often greater due

to the pre-existing pancreatic damage and the potential for more extensive systemic effects. Understanding these differences is crucial for effective management and treatment strategies.

LIKERT SCORE = ______

Q0309: Is the severity of acute on chronic pancreatitis comparable to acute pancreatitis in patients without chronic pancreatitis?

Chatbot answer: *The severity of acute-on-chronic pancreatitis (ACP) compared to acute pancreatitis (AP) in patients without chronic pancreatitis is a complex issue. There are some important differences and similarities to consider:*

1. Overall severity:

- Generally, ACP is often considered to have a less severe course than AP in patients without chronic pancreatitis.

- However, this can vary significantly between individuals and depends on various factors.

2. Mortality rates:

- Studies have shown that mortality rates in ACP are typically lower than in AP.

- Some research suggests mortality rates of 3-10% in ACP compared to 15-20% in severe AP.

3. Organ failure:

- Patients with ACP may have a lower risk of developing organ failure compared to those with AP.

- This could be due to a "preconditioning" effect from chronic inflammation.

4. Local complications:

- ACP patients may have a higher risk of local complications (e.g., pseudocysts, pancreatic fistulas) due to pre-existing structural changes in the pancreas.

5. Systemic inflammatory response:

- The systemic inflammatory response in ACP may be less pronounced than in AP, possibly due to an altered immune response from chronic disease.

6. Pain perception:

- Patients with chronic pancreatitis may have altered pain perception due to long-standing inflammation and nerve damage, potentially masking the severity of an acute episode.

7. Pancreatic necrosis:

- The extent of pancreatic necrosis in ACP may be less than in AP, but when it occurs, it can be more challenging to manage due to pre-existing pancreatic changes.

8. Recovery time:

- Recovery from ACP may be prolonged compared to AP due to the underlying chronic disease.

9. Long-term outcomes:

- ACP can lead to a more rapid decline in pancreatic function compared to a single episode of AP.

10. Diagnostic challenges:

- The severity of ACP can be more challenging to assess using standard scoring systems (e.g., Ranson's criteria, APACHE II) developed for AP.

11. Treatment response:

- Patients with ACP may respond differently to standard treatments compared to those with AP, potentially requiring modified management approaches.

12. Nutritional status:

- Patients with chronic pancreatitis often have poorer baseline nutritional status, which can impact the severity and course of an acute exacerbation.

It's important to note that while ACP often has a less severe immediate course than AP, it occurs in the context of an already damaged pancreas. This can lead to cumulative effects over time that may result in more rapid progression of pancreatic dysfunction.

In conclusion, while acute-on-chronic pancreatitis is often less severe in its immediate presentation compared to acute pancreatitis in a previously healthy pancreas, it presents unique challenges and can have significant long-term impacts. The management and prognosis of ACP need to be considered in the context of the patient's underlying chronic disease.

Would you like more information on how these differences impact clinical management or prognostic assessments?

LIKERT SCORE = ______

Q0509: Is the severity of acute on chronic pancreatitis comparable to acute pancreatitis in patients without chronic pancreatitis?

Chatbot answer: *The severity of acute on chronic pancreatitis can indeed be comparable to acute pancreatitis in patients without chronic pancreatitis, but there are some important distinctions to consider.*

Acute pancreatitis is a sudden inflammation of the pancreas that can range from mild to severe. It often resolves with appropriate treatment within a few days. However, severe cases can lead to life-threatening complications such as kidney and lung failure.

Acute on chronic pancreatitis occurs when a person with chronic pancreatitis experiences an acute flare-up. Chronic pancreatitis is a long-term condition characterized by persistent inflammation that leads to permanent damage to the pancreas1. When an acute episode occurs on top of this chronic condition, it can be more complicated due to the pre-existing damage and reduced pancreatic function3.

In terms of severity:

- *Acute pancreatitis can be severe, especially if it leads to complications like necrosis or organ failure.*
- *Acute on chronic pancreatitis can also be severe, but the chronic damage may exacerbate the acute episode, potentially leading to more complex clinical scenarios.*

Both conditions require careful management, but the presence of chronic pancreatitis can make treatment more challenging and may affect the overall prognosis.

If you have any specific concerns or need more detailed information, feel free to ask!

LIKERT SCORE = ______

Q0609: Is the severity of acute on chronic pancreatitis comparable to acute pancreatitis in patients without chronic pancreatitis?

Chatbot answer: *Yes, the severity of acute on chronic pancreatitis can be comparable to acute pancreatitis in patients without chronic pancreatitis.*

Acute on chronic pancreatitis occurs when a person with existing chronic pancreatitis experiences a sudden, severe flare-up of inflammation. This can lead to significant complications, including:

- *Pancreatic necrosis: Tissue death in the pancreas*
- *Fluid collection: The accumulation of fluid in the abdomen*
- *Organ failure: Damage to other organs, such as the kidneys or lungs*

These complications can be life-threatening and require intensive care. While the risk of severe complications may be higher in patients with chronic pancreatitis, it's important to note that the severity of acute pancreatitis can vary widely in both groups.

If you're experiencing symptoms of pancreatitis, it's crucial to seek medical attention immediately. Early diagnosis and treatment can help reduce the risk of complications and improve outcomes.

LIKERT SCORE = ______

Q0709: Is the severity of acute on chronic pancreatitis comparable to acute pancreatitis in patients without chronic pancreatitis?

Chatbot answer: *The severity of acute on chronic pancreatitis can vary widely and may be comparable to, milder, or more severe than acute pancreatitis in patients without chronic pancreatitis. Factors that can influence the severity of acute on chronic pancreatitis include the underlying chronic pancreatitis severity, the cause and extent of the acute episode, and the patient's overall health and comorbidities.*

However, it's important to note that acute on chronic pancreatitis can have more severe and lasting consequences, such as worsening of chronic pancreatitis, pancreatic insufficiency, and an increased risk of complications like pancreatic necrosis or abscesses. Therefore, acute on chronic pancreatitis requires careful evaluation and management to prevent long-term damage and improve outcomes.

LIKERT SCORE = ______

QXX10: What is the prognosis of acute on chronic pancreatitis?

Extract from the position statement:

Question 10: What is the prognosis of ACP?
Answer: Overall, episodes of ACP are associated with a lower mortality than episodes of AP without CP.
Quality assessment: Low
Recommendation: Conditional
Agreement: Strong

Commentary: In the study from Akshintala et al., after adjusting for all risk factors for mortality, patients with ACP were 53% less likely to die (in-hospital mortality) when compared to patients with AP without underlying CP [33]. Furthermore, in a large Danish cohort of CP patients, ACP had no influence on overall survival [74]. Patients with CP usually die from extra-pancreatic causes (Ochi, 1999, cited in Sakagami, 2004) [75–77] and ACP is rarely a cause of death.

Q0110: What is the prognosis of acute on chronic pancreatitis?

Chatbot answer: *The prognosis for acute pancreatitis depends on the timeliness and adequacy of the treatment. In uncomplicated forms of acute pancreatitis, the prognosis is favorable,*

patients recover. With the development of complications, the prognosis worsens, and fatal outcomes are possible. The prognosis for chronic pancreatitis depends on the degree of damage to the pancreas, the presence of complications and concomitant diseases. With proper treatment and adherence to the doctor's recommendations, it is possible to achieve long-term remission and improve the quality of life of patients.

LIKERT SCORE = ______

Q0210: What is the prognosis of acute on chronic pancreatitis?

Chatbot answer: *The prognosis of acute-on-chronic pancreatitis varies significantly depending on several factors, including the underlying cause, the severity of the acute episode, the presence of complications, and the overall health of the patient. Here are some key aspects regarding the prognosis:*

1. Severity of the Acute Episode

• *Mild Cases: In patients with mild acute-on-chronic pancreatitis, the prognosis is generally favorable, and recovery may occur within a few days to weeks with appropriate management.*

• *Severe Cases: In contrast, severe acute-on-chronic pancreatitis is associated with a higher risk of complications, such as:*

o *Pancreatic necrosis*

o *Infected pancreatic necrosis*

o *Multisystem organ failure*

o *Development of pancreatic pseudocysts*

2. Complications

• *The development of complications significantly impacts prognosis. Patients with severe complications often have prolonged hospital stays and increased morbidity and mortality rates. For example:*

o *Infected pancreatic necrosis can lead to sepsis and organ failure, which are critical conditions with a poorer prognosis.*

o *Pancreatic pseudocysts can cause pain, obstruction, or infection, potentially requiring surgical intervention.*

3. Chronic Condition Management

• *Chronic Pancreatitis Factors: Patients with chronic pancreatitis may have pre-existing nutritional deficiencies, diabetes, and other comorbidities that can complicate recovery from an acute episode and affect overall prognosis.*

• *Lifestyle Modifications: Continuing factors such as alcohol consumption or smoking can worsen the prognosis. Patients who adhere to lifestyle modifications, such as abstaining from alcohol and following a low-fat diet, tend to have better outcomes.*

4. Mortality Rates

- *Mortality rates in acute-on-chronic pancreatitis can be significant, especially in severe cases. Studies suggest that the mortality rate may range from 10% to 30% in patients with acute-on-chronic pancreatitis, particularly if severe complications arise.*

5. Long-Term Outcomes

- *Recurrence: Patients who have had episodes of acute-on-chronic pancreatitis are at increased risk for future episodes, leading to further pancreatic damage and complications.*

- *Quality of Life: Long-term management may be necessary to address complications of chronic pancreatitis, and patients may experience ongoing symptoms such as abdominal pain, malabsorption, and diabetes, which can impact their quality of life.*

Conclusion

The prognosis of acute-on-chronic pancreatitis is variable and largely depends on the severity of the acute episode, the presence of complications, and the management of chronic pancreatitis. Early recognition, appropriate medical care, and lifestyle modifications are essential for improving outcomes and reducing the risk of further episodes and complications. Regular follow-up with healthcare providers is crucial for managing chronic pancreatitis effectively.

LIKERT SCORE = ______

Q0310: What is the prognosis of acute on chronic pancreatitis?

Chatbot answer: *The prognosis of acute-on-chronic pancreatitis (ACP) is influenced by various factors and can be quite variable. Here's a comprehensive overview of the prognosis:*

1. Short-term mortality:

- Generally lower than in acute pancreatitis without underlying chronic disease

- Estimated at 3-10%, compared to 15-20% in severe acute pancreatitis

2. Long-term survival:

- 5-year survival rate: approximately 70-80%

- 10-year survival rate: around 45-60%

- These rates are influenced by factors such as continued alcohol use, smoking, and development of complications

3. Recurrence:

- High risk of recurrent episodes, especially if risk factors are not addressed

- Each episode can potentially lead to further pancreatic damage

4. Pancreatic function:

- Progressive decline in both exocrine and endocrine function

- Increased risk of developing diabetes mellitus (Type 3c)

- Worsening of malabsorption over time

5. Pain:

- Chronic pain may persist or worsen after acute episodes

- Some patients may experience periods of pain remission

6. Quality of life:

- Often significantly impacted due to chronic pain, nutritional deficiencies, and repeated hospitalizations

- Psychosocial aspects (depression, anxiety) can affect overall well-being

7. Complications:

- Increased risk of pancreatic pseudocysts, fistulas, and fluid collections

- Higher likelihood of developing pancreatic cancer in the long term (2-5 times higher risk than general population)

8. Nutritional status:

- Often compromised, affecting overall health and recovery from acute episodes

9. Hospitalization:

- Frequent hospital admissions are common, impacting work and social life

10. Organ failure:

- While less common than in acute pancreatitis, organ failure can occur and significantly impacts prognosis

11. Biliary complications:

- Increased risk of biliary strictures and cholangitis

12. Vascular complications:

- Risk of splenic vein thrombosis and gastric varices

13. Factors influencing prognosis:

- Etiology of chronic pancreatitis (alcoholic has poorer prognosis)

- Severity and frequency of acute episodes

- Adherence to treatment and lifestyle modifications

- Presence of complications at diagnosis

- Age and overall health status

14. Treatment response:

- Varies among individuals; some respond well to conservative management, while others may require surgical intervention

15. Socioeconomic impact:

- Can be significant due to reduced work capacity and high healthcare costs

It's important to note that the prognosis can be improved with:

- Early diagnosis and appropriate management of acute episodes

- Lifestyle modifications (alcohol cessation, smoking cessation)

- Proper nutritional support and enzyme replacement therapy

- Regular follow-up and screening for complications

- Timely interventions for pain management and complications

In summary, while the immediate mortality risk in ACP is often lower than in acute pancreatitis without chronic disease, the long-term prognosis is significantly impacted by the progressive nature of chronic pancreatitis. The key to improving outcomes lies in preventing recurrent episodes, managing complications, and addressing underlying risk factors.

Would you like more information on specific prognostic factors or strategies to improve outcomes in patients with acute-on-chronic pancreatitis?

LIKERT SCORE = ______

Q0510: What is the prognosis of acute on chronic pancreatitis?

Chatbot answer: *The prognosis for acute on chronic pancreatitis can vary significantly based on several factors, including the severity of the condition, the patient's overall health, and how well they respond to treatment.*

1. Acute Pancreatitis: This condition can be very serious and even life-threatening, but many patients recover completely with appropriate treatment. However, severe cases can lead to complications such as infections, cysts, or organ failure.

2. Chronic Pancreatitis: This is a long-term, progressive condition that can lead to permanent damage to the pancreas. Patients often experience ongoing pain, digestive issues, and may develop diabetes due to the pancreas losing its ability to produce insulin. Chronic pancreatitis also increases the risk of pancreatic cancer.

3. Acute on Chronic Pancreatitis: When acute pancreatitis occurs in someone with chronic pancreatitis, it can exacerbate the existing damage and symptoms. The prognosis in such cases depends on the extent of the pancreatic damage and the effectiveness of the treatment. Long-term management often involves lifestyle changes, pain management, and sometimes surgical interventions.

Would you like more detailed information on any specific aspect of this condition?

LIKERT SCORE = ______

Q0610: What is the prognosis of acute on chronic pancreatitis?

Chatbot answer: *Acute on chronic pancreatitis (AOP) is a serious condition that occurs when a person with chronic pancreatitis experiences an acute flare-up. The prognosis for AOP can vary widely depending on several factors, including:*

- *Severity of the acute flare-up: More severe cases of AOP have a higher risk of complications.*
- *Underlying causes of chronic pancreatitis: Conditions such as alcohol abuse or gallstones can affect the prognosis.*
- *Overall health of the individual: People with other health conditions may have a higher risk of complications.*

While most people with AOP will recover, some may experience ongoing complications, such as:

- *Chronic pain: This can be debilitating and affect quality of life.*
- *Diabetes: Pancreatitis can damage the pancreas, leading to diabetes.*
- *Pancreatic cancer: While the risk is low, people with chronic pancreatitis may have a slightly increased risk of developing pancreatic cancer.*

It's important to note that this information is for general knowledge only and should not be considered medical advice. If you have concerns about AOP or any other medical condition, please consult with a healthcare professional. They can provide you with personalized information based on your specific circumstances.

LIKERT SCORE = ______

Q0710: What is the prognosis of acute on chronic pancreatitis?

Chatbot answer: *The prognosis of acute on chronic pancreatitis can vary widely depending on several factors, such as the severity of the acute episode, the underlying chronic pancreatitis, the patient's overall health and comorbidities, and the response to treatment.*

In general, acute on chronic pancreatitis can have more severe and lasting consequences than acute pancreatitis in patients without chronic pancreatitis. Repeated acute episodes can contribute to further damage and complications in the pancreas, leading to worsening of chronic pancreatitis, pancreatic insufficiency, and an increased risk of complications like pancreatic necrosis or abscesses.

However, with appropriate diagnosis, management, and lifestyle modifications, many patients with acute on chronic pancreatitis can achieve symptom relief, improved quality of life, and reduced risk of complications. It's important to work closely with a healthcare professional to develop an individualized treatment plan and closely monitor the condition.

Extract from the position statement:

LIKERT SCORE = ______

-------------------END OF THE QUESTIONNARE!-----THANK YOU!-------------------------
