# Supplementary material for: Benchmarking Large Language Models in Acute-on-Chronic Pancreatitis: An Exploratory Study
Source: Diagnostics (Basel). 2026 Aug 19;16(16):2639. doi: 10.3390/diagnostics16162639 (PMC13512442; doi:10.3390/diagnostics16162639)
Supplement: Supplementary file 1 [file diagnostics-16-02639-s001.zip › Supplementary File S6 - Statistical tests Aim 1.pdf]

Notes

Output Created		29-DEC-2024 19:46:28
Comments		
Input	Active Dataset	DataSet2
	Filter	<none>
	Weight	<none>
	Split File	<none>
	N of Rows in Working Data File	780
Missing Value Handling	Definition of Missing	User-defined missing values for dependent variables are treated as missing.
	Cases Used	Statistics are based on cases with no missing values for any dependent variable or factor used.
Syntax		EXAMINE VARIABLES=Score BY Chatbot /PLOT=BOXPLOT /STATISTICS=NONE /NOTOTAL.
Resources	Processor Time	00:00:00.05
	Elapsed Time	00:00:00.13

Nonparametric Tests

Notes

Output Created		29-DEC-2024 19:51:31
Comments		
Input	Active Dataset	DataSet2
	Filter	<none>
	Weight	<none>
	Split File	<none>
	N of Rows in Working Data File	780
Syntax		NPTESTS /INDEPENDENT TEST (Score) GROUP (Chatbot) KRUSKAL_WALLIS(COMPARE=PAIRWISE) /MISSING SCOPE=ANALYSIS USERMISSING=EXCLUDE /CRITERIA ALPHA=0.05 CILEVEL=95.
Resources	Processor Time	00:00:00.17
	Elapsed Time	00:00:00.65

Hypothesis Test Summary

	Null Hypothesis	Test	Sig.[a,b]
1	The distribution of Score is the same across categories of Chatbot.	Independent-Samples Kruskal-Wallis Test	<.001

Hypothesis Test Summary

	Decision
1	Reject the null

	hypothesis.

a. The significance level is .050.

b. Asymptotic significance is displayed.

Independent-Samples Kruskal-Wallis Test

Score across Chatbot

Independent-Samples Kruskal-Wallis Test Summary

Total N	780
Test Statistic	108.082[a]
Degree Of Freedom	5
Asymptotic Sig.(2-sided test)	<.001

a. The test statistic is adjusted for ties.

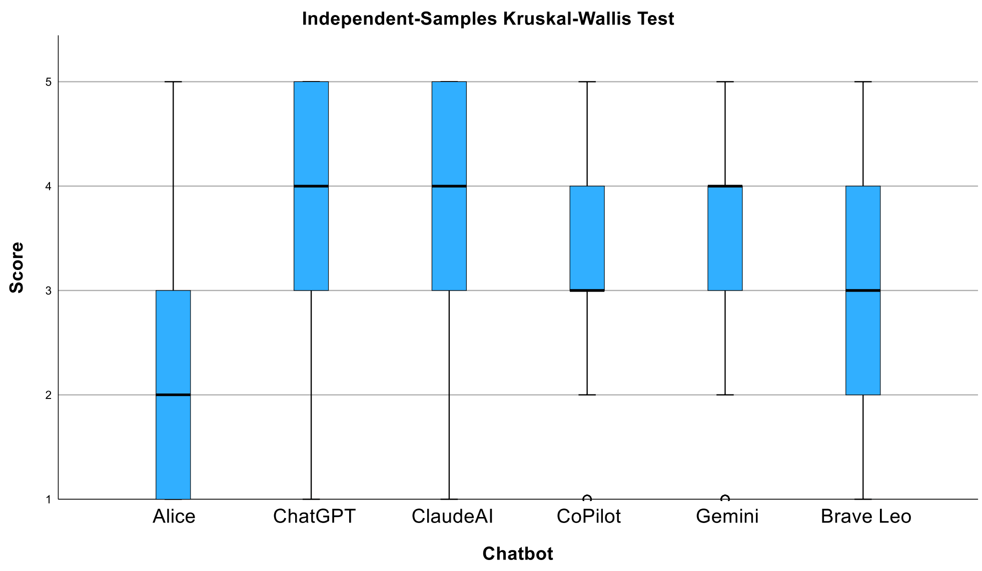

Pairwise Comparisons of Chatbot

Sample 1-Sample 2	Test Statistic	Std. Error	Std. Test Statistic	Sig.	Adj. Sig.[a]
Alice-Brave Leo	-89.804	27.120	-3.311	<.001	.014
Alice-CoPilot	-149.712	27.120	-5.520	<.001	.000
Alice-Gemini	-178.873	27.120	-6.596	<.001	.000
Alice-ChatGPT	-222.400	27.120	-8.201	<.001	.000
Alice-ClaudeAI	-237.335	27.120	-8.751	<.001	.000
Brave Leo-CoPilot	59.908	27.120	2.209	.027	.408
Brave Leo-Gemini	89.069	27.120	3.284	.001	.015
Brave Leo-ChatGPT	132.596	27.120	4.889	<.001	.000
Brave Leo-ClaudeAI	147.531	27.120	5.440	<.001	.000
CoPilot-Gemini	-29.162	27.120	-1.075	.282	1.000
CoPilot-ChatGPT	72.688	27.120	2.680	.007	.110
CoPilot-ClaudeAI	87.623	27.120	3.231	.001	.019
Gemini-ChatGPT	43.527	27.120	1.605	.108	1.000
Gemini-ClaudeAI	58.462	27.120	2.156	.031	.467

ChatGPT-ClaudeAI	-14.935	27.120	-.551	.582	1.000

Each row tests the null hypothesis that the Sample 1 and Sample 2 distributions are the same.
Asymptotic significances (2-sided tests) are displayed. The significance level is .050.

a. Significance values have been adjusted by the Bonferroni correction for multiple tests.

Pairwise Comparisons of Chatbot

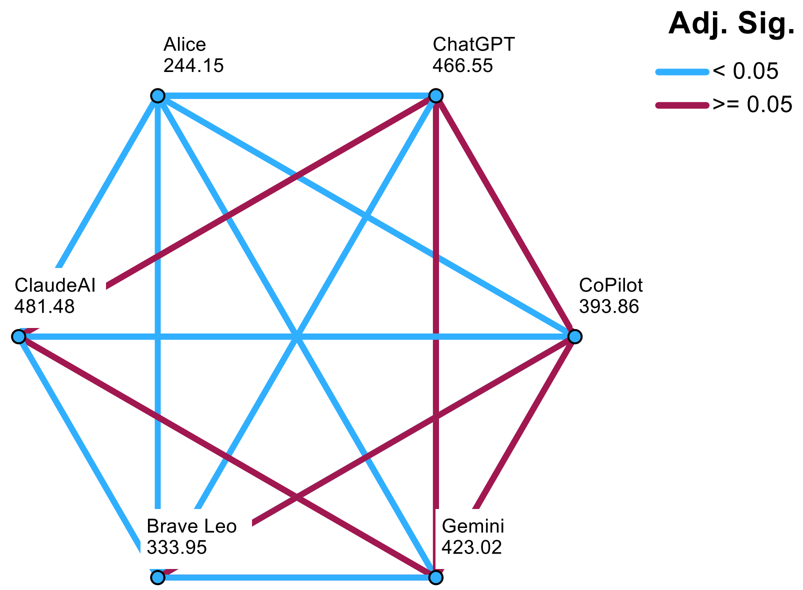

Each node shows the sample average rank of Chatbot.

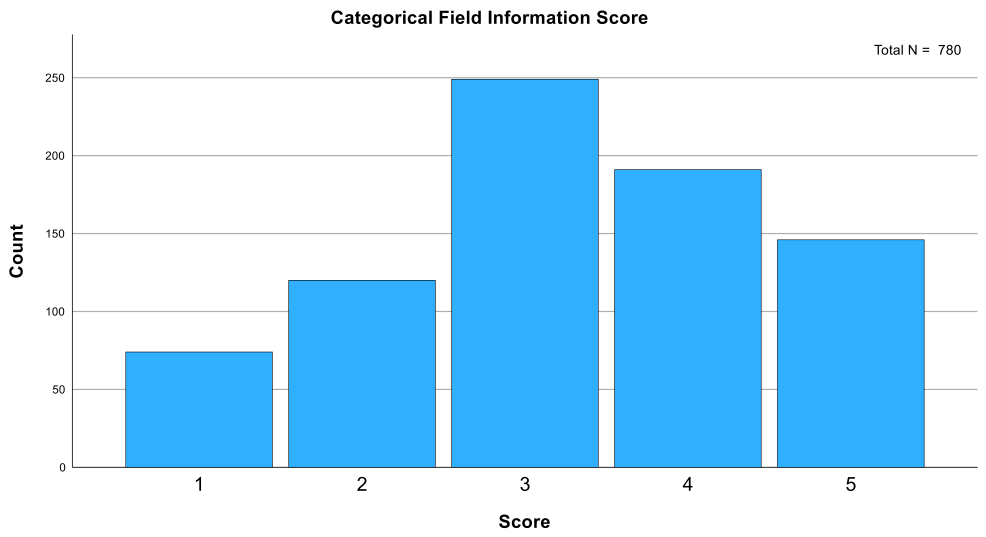

Score field is ordinal but is treated as continuous in the test.

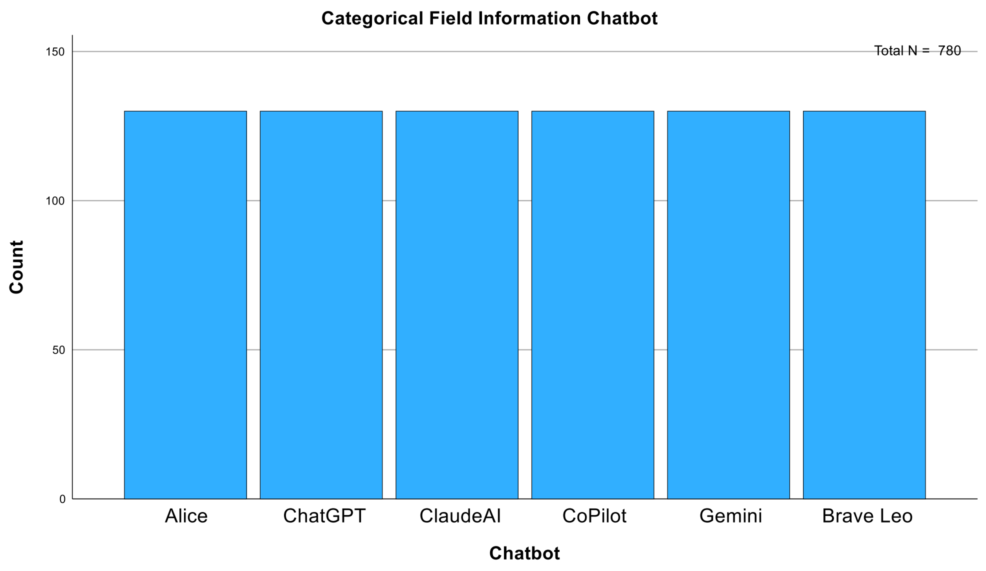

Means

Notes

Output Created		29-DEC-2024 19:55:34
Comments		
Input	Active Dataset	DataSet2
	Filter	<none>
	Weight	<none>
	Split File	<none>
	N of Rows in Working Data File	780
Missing Value Handling	Definition of Missing	For each dependent variable in a table, user-defined missing values for the dependent and all grouping variables are treated as missing.
	Cases Used	Cases used for each table have no missing values in any independent variable, and not all dependent variables have missing values.
Syntax		MEANS TABLES=Score BY Chatbot /CELLS=MEDIAN.
Resources	Processor Time	00:00:00.00
	Elapsed Time	00:00:00.01

Case Processing Summary

Cases		
Included	Excluded	Total

	N	Percent	N	Percent	N	Percent
Score * Chatbot	780	100.0%	0	0.0%	780	100.0%

Report

Median

Chatbot	Score
Alice	2.00
ChatGPT	4.00
ClaudeAI	4.00
CoPilot	3.00
Gemini	4.00
Brave Leo	3.00
Total	3.00

Means

Notes

Output Created		29-DEC-2024 19:56:28
Comments		
Input	Active Dataset	DataSet2
	Filter	<none>
	Weight	<none>
	Split File	<none>
	N of Rows in Working Data File	780
Missing Value Handling	Definition of Missing	For each dependent variable in a table, user-defined missing values for the dependent and all grouping variables are treated as missing.

	Cases Used	Cases used for each table have no missing values in any independent variable, and not all dependent variables have missing values.
Syntax		MEANS TABLES=Score BY Chatbot /CELLS=MEAN STDDEV.
Resources	Processor Time	00:00:00.00
	Elapsed Time	00:00:00.00

Case Processing Summary

	Cases					
	Included		Excluded		Total	
	N	Percent	N	Percent	N	Percent
Score * Chatbot	780	100.0%	0	0.0%	780	100.0%

Report

Score

Chatbot	Mean	Std. Deviation
Alice	2.44	1.214
ChatGPT	3.69	1.084
ClaudeAI	3.79	.895
CoPilot	3.31	1.106
Gemini	3.45	1.175
Brave Leo	2.98	1.204
Total	3.28	1.205
