# Supplementary material for: Benchmarking Large Language Models in Acute-on-Chronic Pancreatitis: An Exploratory Study
Source: Diagnostics (Basel). 2026 Aug 19;16(16):2639. doi: 10.3390/diagnostics16162639 (PMC13512442; doi:10.3390/diagnostics16162639)
Supplement: Supplementary file 1 [file diagnostics-16-02639-s001.zip › Supplementary File S7 - Statistical tests Aim 2.pdf]

Explore

Notes

Output Created		29-DEC-2024 21:17:32
Comments		
Input	Active Dataset	DataSet3
	Filter	<none>
	Weight	<none>
	Split File	<none>
	N of Rows in Working Data File	6
Missing Value Handling	Definition of Missing	User-defined missing values for dependent variables are treated as missing.
	Cases Used	Statistics are based on cases with no missing values for any dependent variable or factor used.
Syntax		EXAMINE VARIABLES=AvgLikert Avgresponse<br>/PLOT BOXPLOT HISTOGRAM NPPLOT<br>/COMPARE GROUPS<br>/STATISTICS DESCRIPTIVES EXTREME<br>/CINTERVAL 95<br>/MISSING LISTWISE<br>/NOTOTAL.
Resources	Processor Time	00:00:00.20

Elapsed Time	00:00:00.70

Case Processing Summary

	Cases					
	Valid		Missing		Total	
	N	Percent	N	Percent	N	Percent
Avg Likert	6	100.0%	0	0.0%	6	100.0%
Avg response	6	100.0%	0	0.0%	6	100.0%

Descriptives

			Statistic	Std. Error
Avg Likert	Mean		3.2767	.20441
	95% Confidence Interval for Mean	Lower Bound	2.7512	
		Upper Bound	3.8021	
	5% Trimmed Mean		3.2946	
	Median		3.3800	
	Variance		.251	
	Std. Deviation		.50071	
	Minimum		2.44	
	Maximum		3.79	
	Range		1.35	
	Interquartile Range		.87	
	Skewness		-.958	.845
	Kurtosis		.402	1.741
Avg response	Mean		172.750	44.9560
	95% Confidence Interval for Mean	Lower Bound	57.187	
		Upper Bound	288.313	

	5% Trimmed Mean	172.156	
	Median	150.850	
	Variance	12126.227	
	Std. Deviation	110.1191	
	Minimum	44.7	
	Maximum	311.5	
	Range	266.8	
	Interquartile Range	225.0	
	Skewness	.356	.845
	Kurtosis	-1.829	1.741

Extreme Values[a]

			Case Number	Value
Avg Likert	Highest	1	3	3.79
		2	2	3.69
		3	5	3.45
	Lowest	1	1	2.44
		2	6	2.98
		3	4	3.31
Avg response	Highest	1	3	311.5
		2	2	294.8
		3	5	175.5
	Lowest	1	1	44.7
		2	6	83.8
		3	4	126.2

a. The requested number of extreme values exceeds the number of data points. A smaller number of extremes is displayed.

Tests of Normality

Kolmogorov-Smirnov[a]	Shapiro-Wilk

	Statistic	df	Sig.	Statistic	df	Sig.
Avg Likert	.193	6	.200*	.930	6	.580
Avg response	.199	6	.200*	.912	6	.448

*. This is a lower bound of the true significance.

a. Lilliefors Significance Correction

Avg Likert

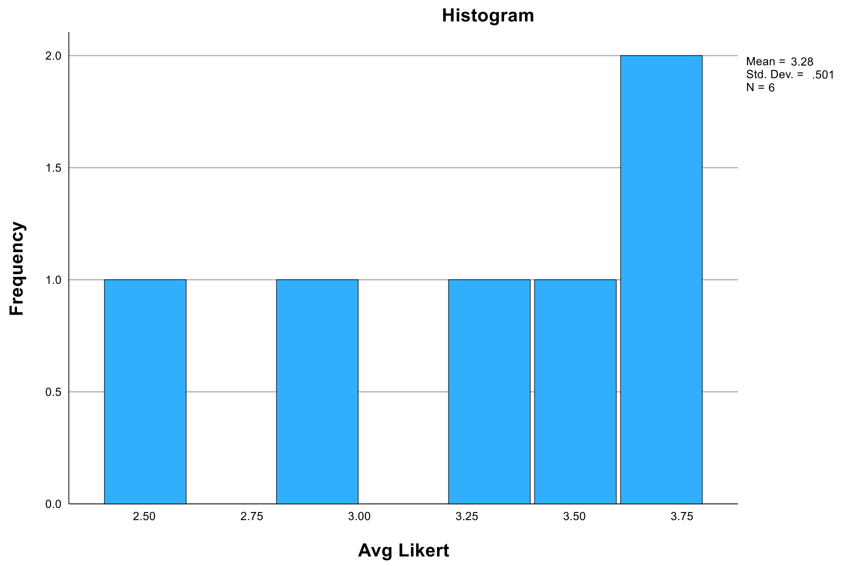

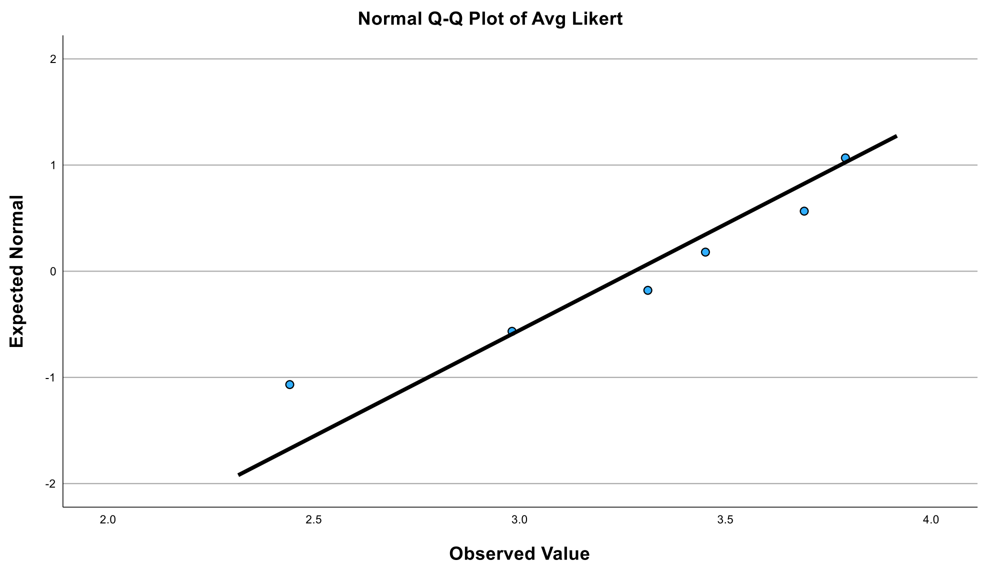
Normal Q-Q Plot of Avg Likert
Expected Normal
2
1
0
-1
-2
2.0
2.5
3.0
3.5
4.0
Observed Value

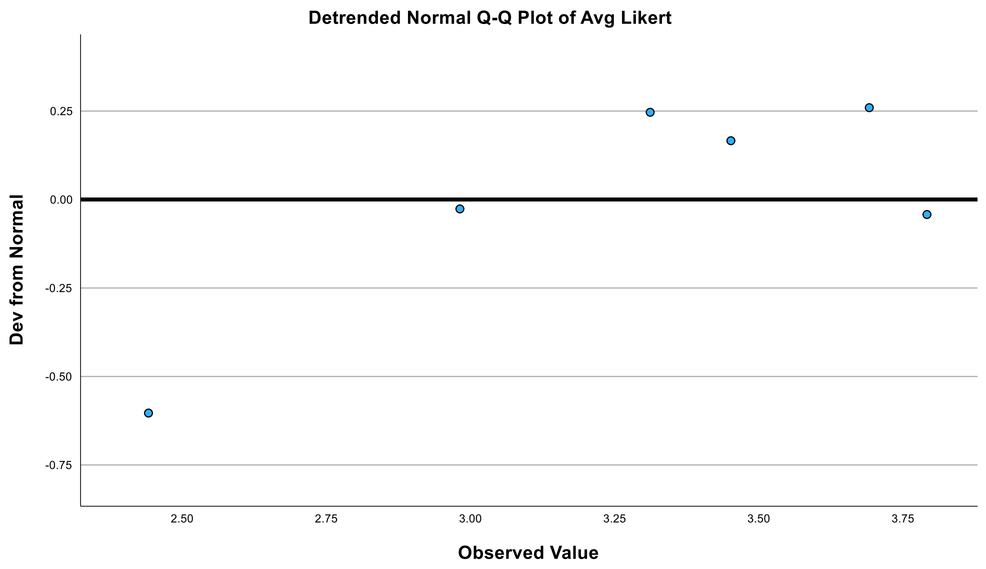
Detrended Normal Q-Q Plot of Avg Likert
Dev from Normal
0.25
0.00
-0.25
-0.50
-0.75
2.50
2.75
3.00
3.25
3.50
3.75
Observed Value

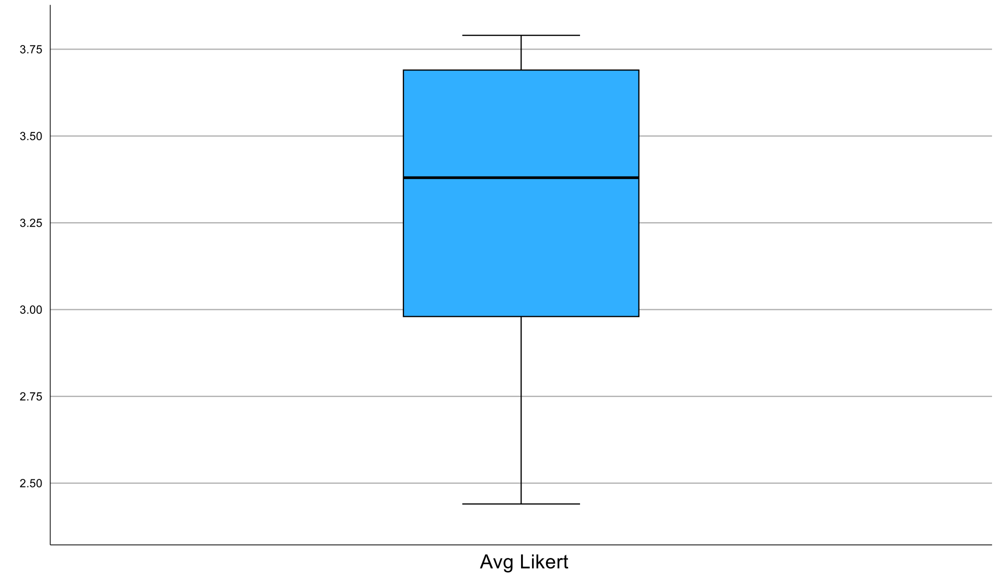

Avg response

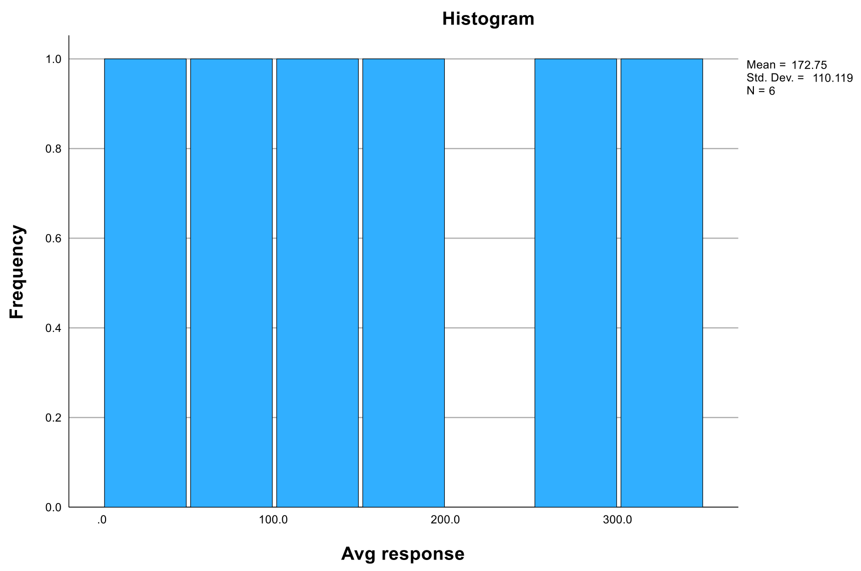
Histogram
Mean = 172.75
Std. Dev. = 110.119
N = 6
Frequency
1.0
0.8
0.6
0.4
0.2
0.0
.0
100.0
200.0
300.0
Avg response

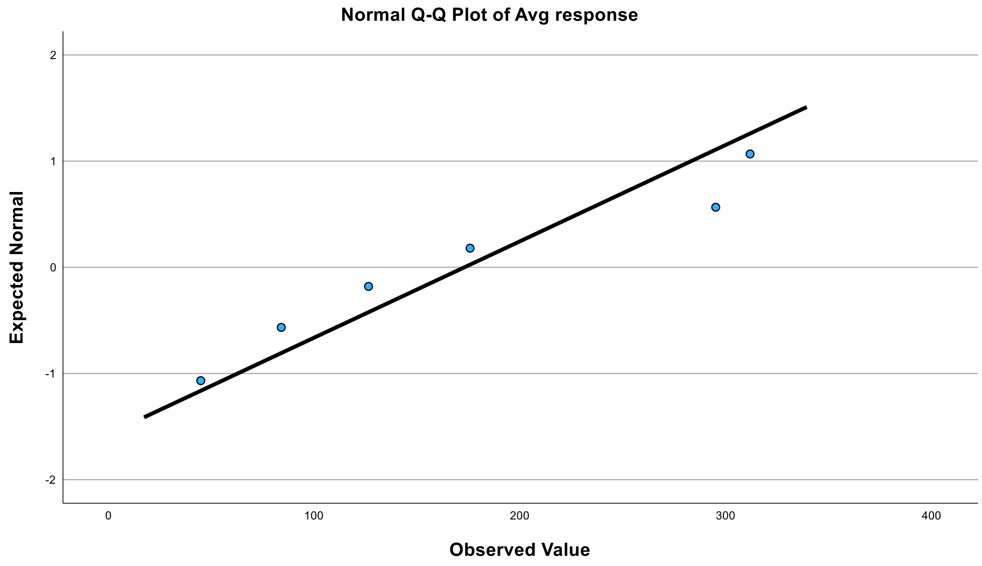
Normal Q-Q Plot of Avg response
Expected Normal
2
1
0
-1
-2
0
100
200
300
400
Observed Value

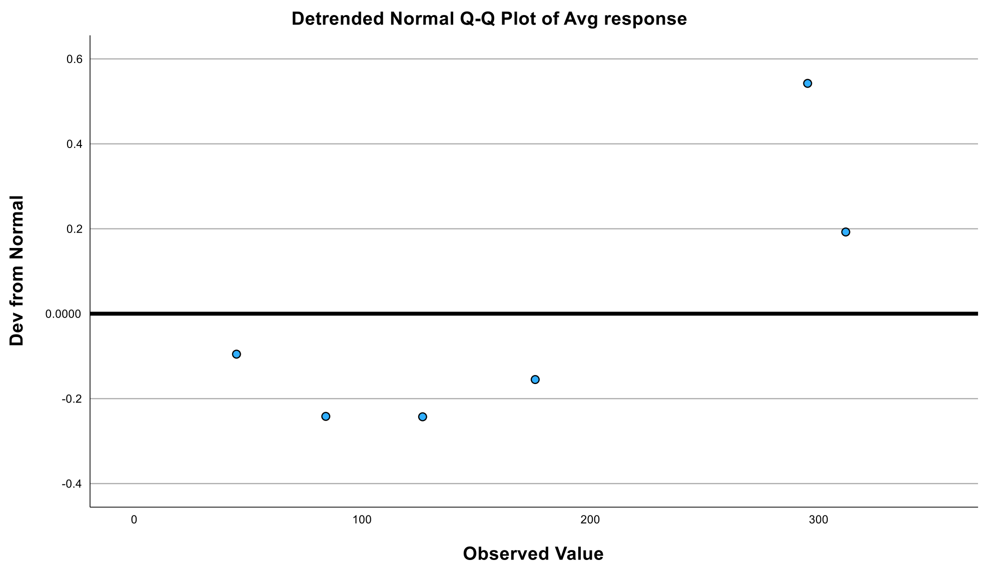
Detrended Normal Q-Q Plot of Avg response
Dev from Normal
0.6
0.4
0.2
0.0000
-0.2
-0.4
0
100
200
300
Observed Value

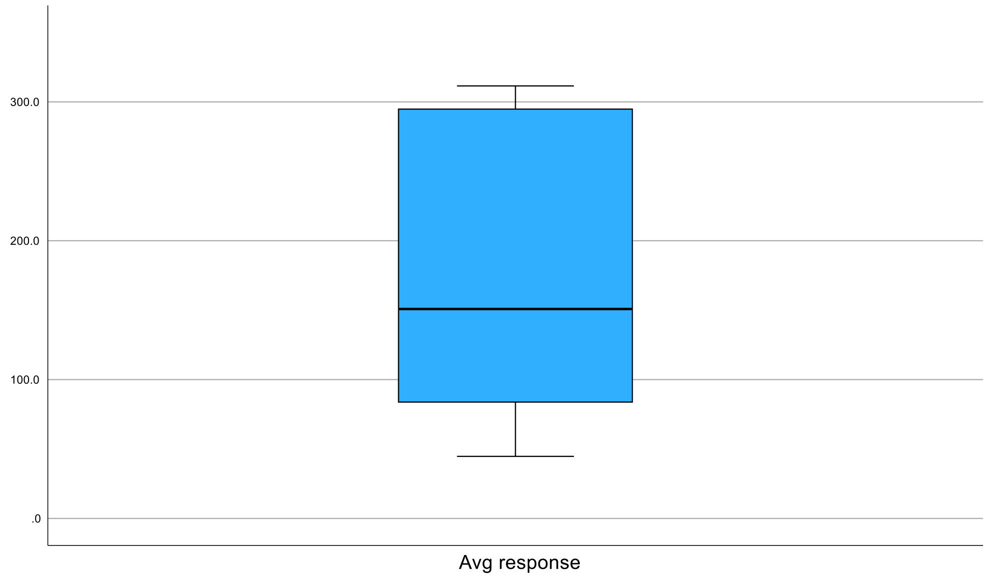
300.0
200.0
100.0
.0
Avg response

Correlations

Notes

Output Created		29-DEC-2024 21:27:48
Comments		
Input	Active Dataset	DataSet3
	Filter	<none>
	Weight	<none>
	Split File	<none>
	N of Rows in Working Data File	6
Missing Value Handling	Definition of Missing	User-defined missing values are treated as missing.
	Cases Used	Statistics for each pair of variables are based on all the cases with valid data for that pair.
Syntax		CORRELATIONS /VARIABLES=AvgLikert Avgresponse /PRINT=TWOTAIL NOSIG FULL /MISSING=PAIRWISE.
Resources	Processor Time	00:00:00.02
	Elapsed Time	00:00:00.04

Correlations

		Avg Likert	Avg response
Avg Likert	Pearson Correlation	1	.922**
	Sig. (2-tailed)		.009

	N	6	6
Avg response	Pearson Correlation	.922**	1
	Sig. (2-tailed)	.009	
	N	6	6

**. Correlation is significant at the 0.01 level (2-tailed).
