# Supplementary material for: Benchmarking Large Language Models in Acute-on-Chronic Pancreatitis: An Exploratory Study
Source: Diagnostics (Basel). 2026 Aug 19;16(16):2639. doi: 10.3390/diagnostics16162639 (PMC13512442; doi:10.3390/diagnostics16162639)

Correlations

Notes

Output Created		29-DEC-2024 22:38:01
Comments		
Input	Active Dataset	DataSet4
	Filter	<none>
	Weight	<none>
	Split File	<none>
	N of Rows in Working Data File	13
Missing Value Handling	Definition of Missing	User-defined missing values are treated as missing.
	Cases Used	Statistics for each pair of variables are based on all the cases with valid data for that pair.
Syntax		CORRELATIONS /VARIABLES=AvgLikert AvgHindex Experienceinpancreatology /PRINT=TWOTAIL NOSIG FULL /MISSING=PAIRWISE.
Resources	Processor Time	00:00:00.02
	Elapsed Time	00:00:00.05

Correlations

		Avg Likert	Avg H-index	Experience in pancreatology

Avg Likert	Pearson Correlation	1	.036	.237
	Sig. (2-tailed)		.907	.435
	N	13	13	13
Avg H-index	Pearson Correlation	.036	1	.836**
	Sig. (2-tailed)	.907		<.001
	N	13	13	13
Experience in pancreatology	Pearson Correlation	.237	.836**	1
	Sig. (2-tailed)	.435	<.001	
	N	13	13	13

**. Correlation is significant at the 0.01 level (2-tailed).

Graph

Notes

Output Created		29-DEC-2024 22:49:18
Comments		
Input	Active Dataset	DataSet4
	Filter	<none>
	Weight	<none>
	Split File	<none>
	N of Rows in Working Data File	13
Syntax		GRAPH /SCATTERPLOT(BIVAR) =Experienceinpancreatology WITH AvgHindex /MISSING=LISTWISE.
Resources	Processor Time	00:00:00.05

Elapsed Time	00:00:00.07

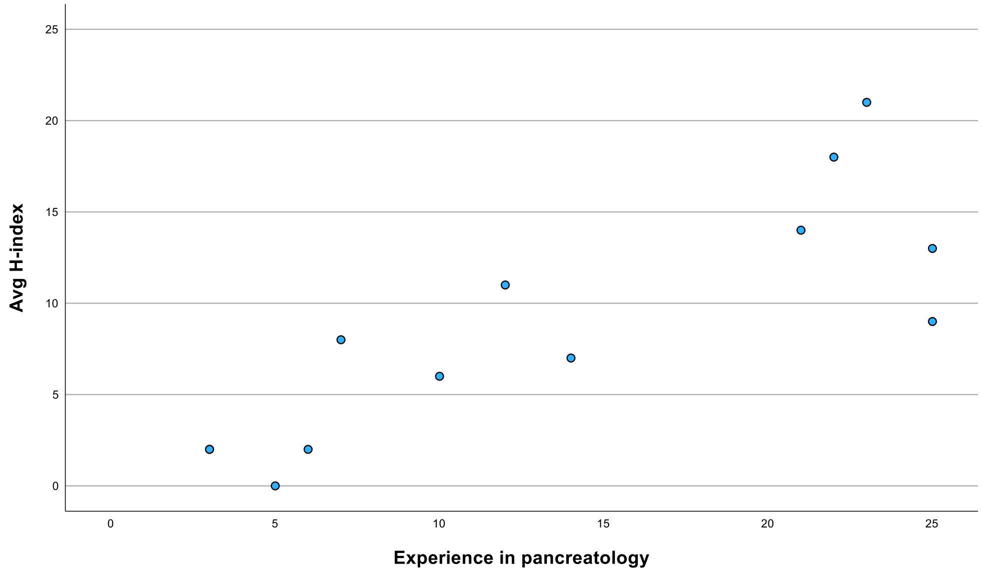

Graph

Notes

Output Created		29-DEC-2024 22:50:02
Comments		
Input	Active Dataset	DataSet4
	Filter	<none>
	Weight	<none>
	Split File	<none>
	N of Rows in Working Data File	13
Syntax		GRAPH /SCATTERPLOT(BIVAR)

		=AvgHindex WITH AvgLikert /MISSING=LISTWISE.
Resources	Processor Time	00:00:00.02
	Elapsed Time	00:00:00.08

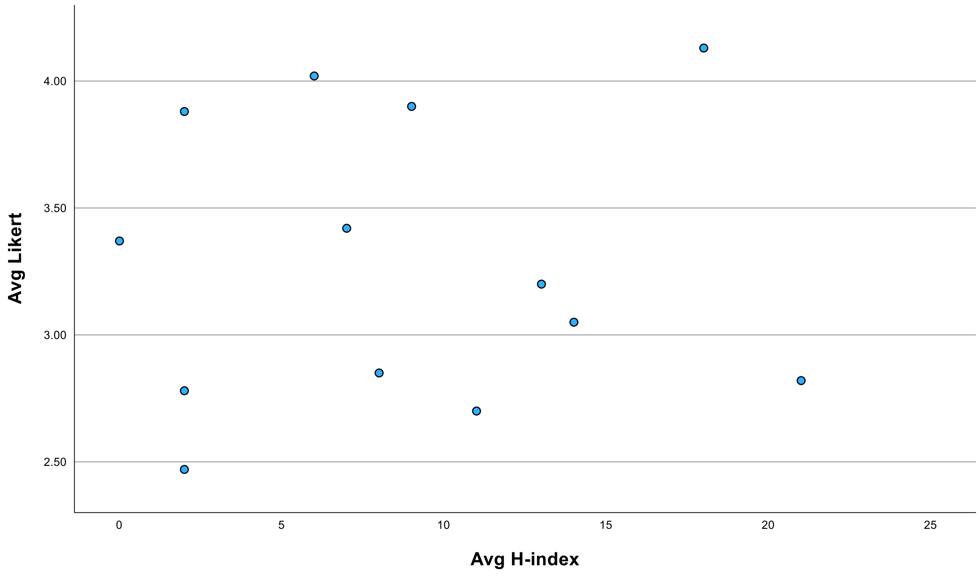

Graph

Notes

Output Created		29-DEC-2024 22:50:27
Comments		
Input	Active Dataset	DataSet4
	Filter	<none>
	Weight	<none>

	Split File	<none>
	N of Rows in Working Data File	13
Syntax		GRAPH /SCATTERPLOT(BIVAR) =Experienceinpancreatology WITH AvgLikert /MISSING=LISTWISE.
Resources	Processor Time	00:00:00.05
	Elapsed Time	00:00:00.10

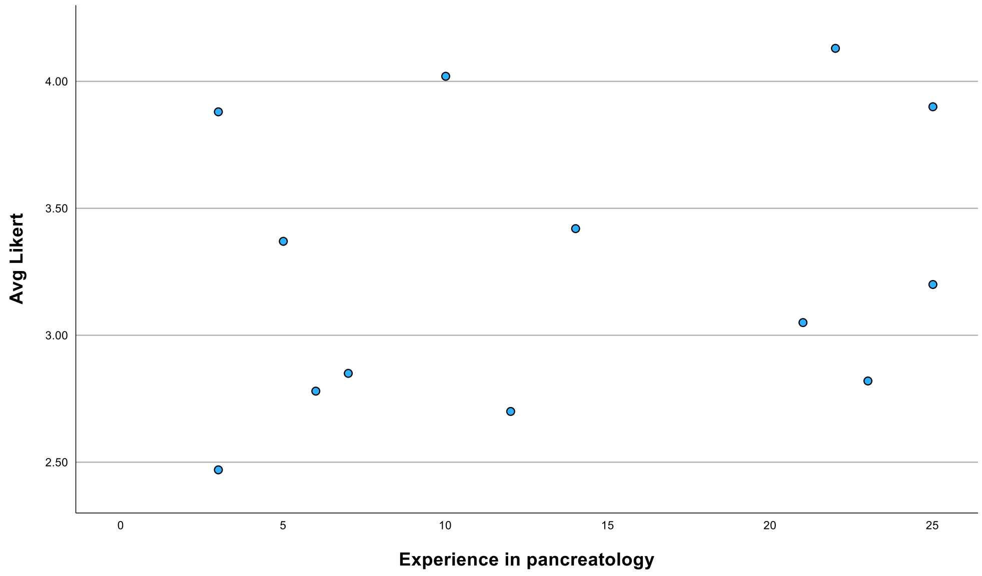

Graph

Notes

Output Created	29-DEC-2024 22:50:57

Comments		
Input	Active Dataset	DataSet4
	Filter	<none>
	Weight	<none>
	Split File	<none>
	N of Rows in Working Data File	13
Syntax		GRAPH /SCATTERPLOT(BIVAR) =AvgHindex WITH AvgLikert /MISSING=LISTWISE.
Resources	Processor Time	00:00:00.02
	Elapsed Time	00:00:00.10

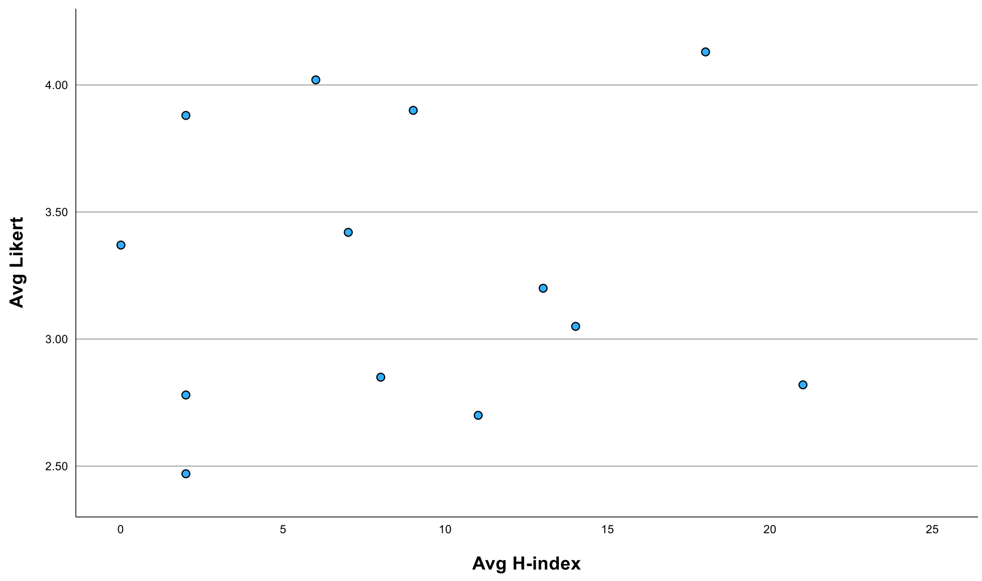

Supplement: Supplementary file 1 [file diagnostics-16-02639-s001.zip › Supplementary File S8 - Statistical tests Aim 3-4.pdf]
