# Supplementary material for: Benchmarking Large Language Models in Acute-on-Chronic Pancreatitis: An Exploratory Study
Source: Diagnostics (Basel). 2026 Aug 19;16(16):2639. doi: 10.3390/diagnostics16162639 (PMC13512442; doi:10.3390/diagnostics16162639)
Supplement: Supplementary file 1 [file diagnostics-16-02639-s001.zip › Supplementary File S9 - Wikipedia 2 oct.pdf]

WIKIPEDIA
The Free Encyclopedia

WIKIPEDIA

List of chatbots

A chatbot is a software application or web interface that is designed to mimic human conversation through text or voice interactions.[1][2][3] Modern chatbots are typically online and use generative artificial intelligence systems that are capable of maintaining a conversation with a user in natural language and simulating the way a human would behave as a conversational partner. Such chatbots often use deep learning and natural language processing, but simpler chatbots have existed for decades.

This **list of chatbots** is a general overview of notable chatbot applications and web interfaces.

General chatbots

Chatbot	Developer	Released	Platform	Technology	License	Notes
**Alexa**	Amazon	2014-11-06	Fire OS, iOS, Android, Linux, Windows, Wear OS	Largely based on a Polish speech synthesizer named Ivona, bought by Amazon in 2013[4][5]	Proprietary	Virtual assistant
**Alice**	Yandex	2017-10-10	Windows, iOS, Android	YandexGPT[6]	?	Virtual assistant
**AliGenie**	Alibaba Group	2017-07-05	?	?	?	Virtual assistant
**Assistant**	Google	2016-05-18	Android, ChromeOS, iOS, iPadOS, KaiOS, Linux, Android TV, Wear OS	?	?	Virtual assistant
**Bixby**	Samsung Electronics	2017-04-21	Android, Tizen, Windows, Wear OS	?	Proprietary, except for open-source components	Virtual assistant
**Braina**	Brainasoft	2014-02-09 or earlier[7]	Windows	Various	Proprietary	Virtual assistant[8][9] and speech-to-text dictation[10] application
**CarynAI**	Caryn Marjorie[11]	2023-05-09	?	BanterAI[12][13][14][15][16]	?	
**Celia**	Huawei	2020-04-27	Android, EMUI, HarmonyOS	Huawei PanGu	Proprietary	Virtual assistant designed for the latest HarmonyOS and Android-based EMUI smartphones that lack Google Services and the Google Assistant[17][18][19][20]
**ChatGPT**	OpenAI	2022-11-30	Web app, iOS, Android	GPT-4, GPT-4o, GPT-4o mini	Proprietary	
**Claude**	Anthropic	2023-03	Web app, iOS	Claude 2.1	Proprietary	
**Cleverbot**	Rollo Carpenter	2008-10	Web app	?	?	Winner of the 2010 Mechanical Intelligence Competition
**Clova**	Naver Corporation	2017-03-01	iOS, Android	?	?	Virtual assistant
**Copilot**	Microsoft	2023-02-07	Web app, Windows, iOS, Android	Microsoft Prometheus, GPT-4	Proprietary	Launched as Bing Chat
**Ernie Bot**	Baidu	2023-03-16	?	?	?	
**Gemini**	Google	2023-03-21	Web app, Android, iOS	Gemini (language model)	Proprietary[21]	
**GigaChat**	Sberbank	2023-04	Web app	?	Proprietary	
**Grok**	xAI	2023-11-04	Web app	?	Proprietary, Apache-2.0 (Grok-1)	Currently under beta testing for those with the premium version of X
**Jabberwacky**	Rollo Carpenter	1997	Web app	?	?	Predecessor to Cleverbot

Chatbot	Developer	Released	Platform	Technology	License	Notes
**Kuki**	Steve Worswick	2013 or earlier[22]	Web app, various social networks	Pandorabots AIML	?	Embodied five-time Loebner Prize winner designed to befriend humans in the metaverse[23][24][25]
**Lenny**	"Mango"[26][27]	2011	?	?	Source-available	An audio bot designed to annoy telemarketers
**Leo**	Brave Software	2023-11-02	Windows, macOS, Linux	LLaMA 2, Claude	?	Included with the Brave desktop browser
**MegaHAL**	Jason Hutchens	1995-04-30	Web app, command line	?	Unlicense	
**SILVIA**	Cognitive Code	2008-01-01	Windows, macOS, iOS, Android	?	Proprietary	Core platform technology
**SimSimi**	ISMaker[28]	2002	Web app, iOS, Android	?	?	
**Siri**	Apple Inc.	2011-10-05	iOS, iPadOS, watchOS, macOS, tvOS, audioOS, visionOS[29][30]	?	?	Virtual assistant
**SpookiTalk**	The Digital Village	1998-04-02	Windows, Classic Mac OS	Based on VelociText[31]	?	Language processor used for non-player characters in *Starship Titanic*
**Q**	Amazon	2023-11-28	Web app	Amazon Titan, Amazon Bedrock,[32] generative pre-trained transformers	?	Developed for enterprise use
**Ultra Hal**	Robert Medeksza	2000-12-04 or earlier[33]	Windows	GPT-3[34]	?	2007 Loebner Prize winner intended to function as a virtual assistant
**Xiaowei**	Tencent	2017-06 (late in the month)	?	?	?	Virtual assistant

Historical chatbots

Chatbot	Developer	Released	Discontinued	Platform	Technology	License	Notes
**Albert One**	Robby Garner	1995	?	The Internet	Based on a multi-faceted approach in natural-language programming	?	1998 and 1999 Loebner Prize winner designed to mimic the way humans make conversations
**Artificial Linguistic Internet Computer Entity**	Richard Wallace	1995-11-23[35]	2013-10-15	?	AIML	Open-source software[36]	Three-time Loebner Prize winner
**Assistant**	Speaktoit	2011-03-01	2016-12-15	Android, iOS, Windows Phone, Windows 8, Windows 10, ChromeOS	?	?	A virtual assistant acquired by Google, unrelated to the Google Assistant

Chatbot	Developer	Released	Discontinued	Platform	Technology	License	Notes
**Charlix**	?	2006-04-17	2010-03-03[37]	Linux	Based on Artificial Linguistic Internet Computer Entity	Open-source software	Desktop virtual assistant
**Cortana**	Microsoft	2014-04-02	2023-08-11	Windows, Windows Phone, iOS, Android, Xbox OS	Tellme Networks, Satori, Microsoft Eva	Proprietary	A deprecated virtual assistant succeeded by Copilot; originally named after character in Xbox *Halo* video game
**Dr. Sbaitso**	Creative Labs	1991-06 or earlier[38]	?	MS-DOS	Speech synthesis	?	Initially released in Singapore
**ELIZA**	Joseph Weizenbaum[39][40]	1964	1967 (stopped development)[41]	?	Pattern matching, MAD-SLIP, lisp-like representation[42]	?	Developed at MIT
**Eugene Goostman**	Vladimir Veselov, Eugene Demchenko, Sergey Ulasen[43][44]	2001	2014-06-07	?	?	?	2012 Turing 100 and 2014 Royal Society Turing test winner some regard as having passed the Turing test
**Evi**	True Knowledge	2012-10	2014-01-23	iOS, Android	?	?	Virtual assistant
**Fred**	Robby Garner	1997-12-01 or earlier[45]	?	?	?	?	
**GooglyMinotaur**	ActiveBuddy (under contract by Capitol Records)	2001-06	2002-03-24	AIM	?	?	ActiveBuddy's first offering,[46][47] specializing in Radiohead-related information[48]
**Infobot**	Kevin Lenzo	1995-06	?	IRC	Perl, factoids	Artistic License	An IRC bot primarily designed to assist with answering FAQs in channels such as *#perl*[49]
**Jeeney AI**	C.J. Jones	2007-02[50]	2010	?	?	?	2009 Chatterbox Challenge winner[51]
**Mark V Shaney**	Rob Pike, Bruce Ellis, Don P. Mitchell	1981	?	Usenet	Markov chain techniques	?	A synthetic user whose postings in the *net.singles* newsgroups were generated based on text from other postings
**Mycroft**	Mycroft team	2015-11-17	2023-01-31	Linux	?	Apache License[52]	Virtual assistant

Chatbot	Developer	Released	Discontinued	Platform	Technology	License	Notes
**PARRY**	Kenneth Colby	1972	?	?	?	?	An early example of a chatbot
**Racter**	Mindscape (publisher)	1984	?	IBM PC compatibles, Apple II, Mac, Amiga	?	?	Was able to generate English-language prose at random[53]
**SmarterChild**	ActiveBuddy	2001-06	2006-10-12	AIM, Windows Live Messenger	?	?	The second bot released by ActiveBuddy[54]
**Sparrow**	Google DeepMind	2022-09	2023-01-12	Web app	Chinchilla	Proprietary	
**Tay**	Microsoft	2016-03-23	2016-03-24	Twitter	?	?	Rapidly decayed into producing racist bigotry after manipulation by online trolls (from 4chan and 8chan); suspended after 16 hours[55][56]
**Verbot**	Avaya	1997	2012 (early in the year)	Microsoft Windows, web app	?	?	An artificial intelligence software development kit[57]
**Viv**	Viv Labs, Inc. (subsidiary of Samsung Electronics)	2016-05-09	2017-10-18	iOS, Android	Integrated into Bixby 2.0	?	Virtual assistant

See also

- The Pile (dataset), public data used to train many research models

References

1. "What is a chatbot?" (http://searchdomino.techtarget.com/sDefinition/0,,sid4_gci935566,00.html). *techtarget.com*. Archived (https://web.archive.org/web/20101102170613/http://searchdomino.techtarget.com/sDefinition/0,,sid4_gci935566,00.html) from the original on 2 November 2010. Retrieved 30 January 2017.
2. Caldarini, Guendalina; Jaf, Sardar; McGarry, Kenneth (2022). "A Literature Survey of Recent Advances in Chatbots" (https://doi.org/10.3390%2Finfo13010041). *Information*. **13** (1). MDPI: 41. arXiv:2201.06657 (https://arxiv.org/abs/2201.06657). doi:10.3390/info13010041 (https://doi.org/10.3390%2Finfo13010041).
3. Adamopoulou, Eleni; Moussiades, Lefteris (2020). "Chatbots: History, technology, and applications" (https://doi.org/10.1016%2Fj.mlwa.2020.100006). *Machine Learning with Applications*. **2**: 100006. doi:10.1016/j.mlwa.2020.100006 (https://doi.org/10.1016%2Fj.mlwa.2020.100006).
4. "Ivona, Alexa, Vika or intelligent girls from Gdańsk" (https://www.sztucznainteligencja.org.pl/en/ivona-alexa-vika-or-intelligent-girls-from-gdansk/). May 22, 2020.
5. "Amazon Gets into Voice Recognition, Buys Ivona Software to Compete Against Apple's Siri" (https://techcrunch.com/2013/01/24/amazon-gets-into-voice-recognition-buys-ivona-software-to-compete-against-apples-siri/). January 24, 2013.
6. "«Яндекс» добавил в «Алису» аналог ChatGPT" (https://www.rbc.ru/technology_and_media/17/05/2023/6464aa4e9a79473a1f0464c1?ysclid=likg8q7hgy78285088) (in Russian). www.rbc.ru. 17 May 2023. Retrieved 2023-06-06.
7. Vladimir A. Fomichov; Alexander A. Razorenov (2014). "The Design of A Natural Language Interface for File System Operations on the basis of a Structured Meanings Model" (https://doi.org/10.1016%2Fj.procs.2014.05.353). *Procedia Computer Science*. **31** (2014). Elsevier: 1005–1011. doi:10.1016/j.procs.2014.05.353 (https://doi.org/10.1016%2Fj.procs.2014.05.353).

8. King, Leo (15 December 2015). "Top 8 virtual personal assistants" (https://web.archive.org/web/20230726225438/https://www.raconteur.net/report/artificial-intelligence-for-business/top-8-virtual-personal-assistants/). *Raconteur*. Archived from the original (https://www.raconteur.net/report/artificial-intelligence-for-business/top-8-virtual-personal-assistants/) on 26 July 2023.
9. Igor Bošnjak; Luka Šaravanja; Eva Čuljak; Željko Stojkić (2021). "Planning and implementation of Digital Assistance System at University of Mostar Learning Factory". *11th Conference on Learning Factories, CLF2021* (2021). SSRN: 3–4. doi:10.2139/ssrn.3858378 (https://doi.org/10.2139%2Fssrn.3858378). S2CID 242604709 (https://api.semanticscholar.org/CorpusID:242604709).
10. "Free Artificial Intelligence (AI) software for your PC" (https://www.zdnet.com/article/free-artificial-intelligence-ai-software-for-your-pc/). *ZDNet*. Retrieved 2024-01-29.
11. "Thousands chatted with this AI 'virtual girlfriend.' Then things got even weirder" (https://www.latimes.com/entertainment-arts/business/story/2023-06-27/influencers-ai-chat-caryn-marjorie). *Los Angeles Times*. 27 June 2023.
12. Lorenz, Taylor (May 13, 2023). "An influencer's AI clone will be your girlfriend for $1 a minute" (https://www.washingtonpost.com/technology/2023/05/13/caryn-ai-technology-gpt-4/). *The Washington Post*.
13. Tolentino, Daysia (May 12, 2023). "Snapchat influencer launches an AI-powered 'virtual girlfriend' to help 'cure loneliness' " (https://www.nbcnews.com/tech/ai-powered-virtual-girlfriend-caryn-marjorie-snapchat-influencer-rcna84180). *NBCNews*.
14. Bibey, Chris (12 May 2023). "Snapchat Sensation Caryn Marjorie's AI Doppelgänger: Your Virtual Girlfriend For $1 Per Minute" (https://finance.yahoo.com/news/snapchat-sensation-caryn-marjories-ai-175416254.html). *Yahoo Finance*.
15. Sternlicht, Alexandra (May 9, 2023). "A 23-year-old Snapchat influencer used OpenAI's technology to create an A.I. version of herself that will be your girlfriend for $1 per minute" (https://fortune.com/2023/05/09/snapchat-influencer-launches-carynai-virtual-girlfriend-bot-openai-gpt4/). *Fortune.com*.
16. Zitser, Josiah (May 11, 2023). "Influencer who created AI version of herself says it's gone rogue and she's working 'around the clock' to stop it saying sexually explicit things" (https://www.insider.com/carynai-ai-virtual-girlfriend-chat-gpt-rogue-filthy-things-influencer-2023-5). *Business Insider*.
17. "Huawei's Celia" (https://web.archive.org/web/20200327130153/https://www.androidauthority.com/huawei-celia-assistant-1098366/%26hl%3Den%3DZA). *Android Authority*. Archived from the original (https://www.androidauthority.com/huawei-celia-assistant-1098366/%26hl%3Den%3DZA) on 27 March 2020.
18. "International Voice Assistant Releases" (https://www.trendhunter.com/trends/celia-voice-assistant). *TrendHunter.com*. Retrieved 2020-06-12.
19. "Huawei Launches Its Own Personal Assistant, That Sounds A Lot Like Apple's" (https://www.androidheadlines.com/2020/03/huawei-celia-launch). *Android Headlines*. 2020-03-26. Retrieved 2020-06-12.
20. "Meet Celia, Huawei's own voice assistant" (https://www.speedmagazine.ph/meet-celia-huaweis-own-voice-assistant/). *Speed Magazine*. 2020-05-20. Retrieved 2020-06-12.
21. David, Emilia (July 20, 2023). "The AI wars might have an armistice deal sooner than expected" (https://www.theverge.com/2023/7/20/23800755/ai-meta-llama-microsoft-chatgpt-interoperability). *The Verge*. Archived (https://web.archive.org/web/20230720150119/https://www.theverge.com/2023/7/20/23800755/ai-meta-llama-microsoft-chatgpt-interoperability) from the original on July 20, 2023. Retrieved July 25, 2023.
22. "Most Loebner Prize wins" (https://www.guinnessworldrecords.com/world-records/603076-most-loebner-prize-wins). *Guinness World Records*. Retrieved 2021-01-28.
23. "New trend report: Into the Metaverse" (https://www.wundermanthompson.com/insight/new-trend-report-into-the-metaverse). *Wunderman Thompson*. Retrieved 2021-11-16.
24. "Kuki Chatbot" (http://www.mitsuku.com/).
25. "Loebner Prize Contest 2013" (http://www.paulmckevitt.com/loebner2013/). People.exeter.ac.uk. 2013-09-14. Retrieved 2013-12-02.
26. Oberhaus, Daniel (November 21, 2018). "The Story of Lenny, the Internet's Favorite Telemarketing Troll" (https://www.vice.com/en_us/article/d3b7na/the-story-of-lenny-the-internets-favorite-telemarketing-troll). *Vice.com*.
27. Bolton, Doug (January 14, 2016). "Meet Lenny – the internet's favourite telemarketer-tricking robot" (http://www.independent.co.uk/life-style/gadgets-and-tech/news/lenny-telemarketer-bot-robot-prank-a6813081.html). *The Independent*. London.
28. "What is Simsimi? All About the iPhone and Android App That Has the World Laughing Out Loud" (http://www.ibtimes.com/articles/285978/20120123/what-simsimi-iphone-android-app.htm). *International Business Times*. 23 January 2012. Archived (https://web.archive.org/web/20120421141736/http://www.ibtimes.com/articles/285978/20120123/what-simsimi-iphone-android-app.htm) from the original on 21 April 2012. Retrieved 25 February 2012.
29. "Use Siri on all your Apple devices" (https://support.apple.com/en-us/HT204389). *support.apple.com*. November 2023.
30. "Google Assistant beats Alexa, Siri" (https://gadgets.ndtv.com/apps/news/google-assistant-beats-alexa-siri-again-in-smartphone-iq-test-2087042). *gadgets.ndtv.com*. August 19, 2019.
31. Richards, Neil (1998). Starship Titanic: The Official Strategy Guide (1st ed.). Three Rivers Press. p. 71-73. ISBN 0-609-80147-3.

32. "What is Amazon Q Business?" (https://docs.aws.amazon.com/amazonq/latest/qbusiness-ug/what-is.html). Retrieved 2024-04-24.
33. "Ultra Hal assistant" (http://www.tribuneindia.com/2000/20001204/login/free.htm). *The Tribune*. 4 December 2000.
34. "Ultra Hal" (https://www.zabaware.com/ultrahal/). Retrieved 2024-02-05.
35. Thompson 2002, p. 2
36. Henderson 2007, pg. 127
37. "Charlix" (https://charlix.sourceforge.net/). Retrieved 2024-02-04.
38. PC Mag Oct 29, 1991 p.67 advertisement (https://books.google.com/books?id=fpQP3e54P-gC&dq=sbaitso+1991&pg=PT80)
39. Berry, David M. (2018). "Weizenbaum, ELIZA and the End of Human Reason". In Baranovska, Marianna; Höltgen, Stefan (eds.). *Hello, I'm Eliza: Fünfzig Jahre Gespräche mit Computern* [*Hello, I'm Eliza: Fifty Years of Conversations with Computers*] (in German) (1st ed.). Berlin: Projekt Verlag. pp. 53–70. ISBN 9783897334670.
40. Weizenbaum, Joseph (1976). *Computer Power and Human Reason: From Judgment to Calculation*. New York: W. H. Freeman and Company. ISBN 0-7167-0464-1.
41. "Alan Turing at 100" (http://news.harvard.edu/gazette/story/2012/09/alan-turing-at-100/). *Harvard Gazette*. 13 September 2012. Retrieved 2016-02-22.
42. Berry, David M. (2023-11-06). "The Limits of Computation: Joseph Weizenbaum and the ELIZA Chatbot" (https://ojs.weizenbaum-institut.de/index.php/wjds/article/view/106). *Weizenbaum Journal of the Digital Society*. **3** (3). doi:10.34669/WI.WJDS/3.3.2 (https://doi.org/10.34669%2FWI.WJDS%2F3.3.2). ISSN 2748-5625 (https://search.worldcat.org/issn/2748-5625).
43. "Computer chatbot 'Eugene Goostman' passes the Turing test" (https://www.zdnet.com/article/computer-chatbot-eugene-goostman-passes-the-turing-test/). ZDNet. 8 June 2014. Archived (https://web.archive.org/web/20140612165756/http://www.zdnet.com/computer-chatbot-eugene-goostman-passes-the-turing-test-7000030312/) from the original on 12 June 2014. Retrieved 8 June 2014.
44. "Turing Test success marks milestone in computing history" (http://www.reading.ac.uk/news-and-events/releases/PR583836.aspx). University of Reading. 8 June 2014. Archived (https://web.archive.org/web/20140608201600/http://www.reading.ac.uk/news-and-events/releases/PR583836.aspx) from the original on 8 June 2014. Retrieved 8 June 2014.
45. L. Caputo, R. Garner, P. Nathan. "FRED, Milton and Barry: the evolution of intelligent agents for the Web" (http://portal.acm.org/citation.cfm?id=304915), Advances in intelligent systems, 1997. *portal.acm.org*
46. "ActiveBuddy Introduces Software to Create and Deploy Interactive Agents for Text Messaging; ActiveBuddy Developer Site Now Open: www.BuddyScript.com". *Business Wire*. 2002-07-15.
47. Gibes, Al (2002-03-25). "Circle of buddies grows ever wider". *Las Vegas Review-Journal (Nevada)*.
48. Gibes, Al (2002-03-25). "Circle of buddies grows ever wider". *Las Vegas Review-Journal (Nevada)*.
49. Lenzo, Kevin (Summer 1998). "Infobots and Purl" (http://www.foo.be/docs/tpj/issues/vol3_2/tpj0302-0002.html). *The Perl Journal*. **3** (2). Retrieved 2010-07-26.
50. Chatbot Jeeney A.I. (https://www.chatbots.org/chatterbot/jeeney_a.i/), chatbots.org (Retrieved 21 February 2024).
51. Ehab El-agizy, (June 30 2009) Chatterbox Challenge 2009 Winners (http://www.chatbots.org/awards/announcement/chatterbox_challenge_2009_winners/), chatbots.org
52. "Having the Right license is just as important as having a License" (https://mycroft.ai/blog/right-license/). 28 June 2017.
53. Chamberlain, Bill (1984). *The Policeman's Beard Is Half Constructed* (https://archive.org/details/policemansbeardi0000unse). UbuWeb, Warner Books. ISBN 0-446-38051-2. Retrieved 2009-07-02.
54. "ActiveBuddy Introduces Software to Create and Deploy Interactive Agents for Text Messaging; ActiveBuddy Developer Site Now Open: www.BuddyScript.com". *Business Wire*. 2002-07-15.
55. Tung, Liam (24 March 2016). "Microsoft's Tay AI chatbot goes offline after being taught to be a racist" (https://www.zdnet.com/article/microsofts-tay-ai-chatbot-goes-offline-after-being-taught-to-be-a-racist/). *ZDNet*. "The internet teaches Microsoft a lesson in the dangers of artificial intelligence and public interaction... Microsoft's millennial-talking AI chatbot, Tay.ai, has taken a break from Twitter after humans taught it to parrot a number of inflammatory and racist opinions... Microsoft had launched Tay on Wednesday, aiming it at people aged between 18 and 24 years in the US. But after 16 busy hours of talking on subjects ranging from Hitler to 9/11 conspiracies, Tay has gone quiet."
56. Reeve, Elspeth. "Tay Exposes the Fairy Tales We Tell Ourselves About Racists" (https://newrepublic.com/article/132039/tay-exposes-fairy-tales-tell-racists). *The New Republic*. Retrieved 11 July 2024. "Tay lived for just 16 hours, until Microsoft "became aware of a coordinated effort by some users to abuse Tay's commenting skills" to make her a Nazi. The /pol/ boards on 4chan and 8chan—/pol/ stands for "politically incorrect"—are where that coordination took place."
57. Quittner, Joshua (1997-12-08). "WHAT'S HOT IN BOTS" (https://web.archive.org/web/20070428130357/http://www.time.com/time/magazine/article/0,9171,987519,00.html). Time Magazine. Archived from the original (http://www.time.com/time/magazine/article/0,9171,987519,00.html) on April 28, 2007.

Retrieved from "https://en.wikipedia.org/w/index.php?title=List_of_chatbots&oldid=1248866970"
